# The critical role of dysregulated Hh-FOXM1-TPX2 signaling in human hepatocellular carcinoma cell proliferation

**DOI:** 10.1186/s12964-020-00628-4

**Published:** 2020-07-28

**Authors:** Yiting Wang, Hailong Wang, Zhengwei Yan, Guohua Li, Guohui Hu, Hong Zhang, Dengliang Huang, Yao Wang, Xiang Zhang, Yehong Yan, Quqin Lu, Minzhang Cheng, Shiwen Luo

**Affiliations:** 1 Center for Experimental Medicine, the First Affiliated Hospital of Nanchang University, 17 Yongwai Street, Donghu District, Nanchang, 330006 Jiangxi China; 2 Jiangxi Key Laboratory of Molecular Diagnostics and Precision Medicine, 17 Yongwai Street, Donghu District, Nanchang, 330006 Jiangxi China; 3 Department of Gastroenterology, the First Affiliated Hospital of Nanchang University, 17 Yongwai Street, Donghu District, Nanchang, 330006 Jiangxi China; 4 Department of General Surgery, the First Affiliated Hospital of Nanchang University, 17 Yongwai Street, Nanchang, 330006 Jiangxi China; 5 Department of Epidemiology & Biostatistics, School of Public Health, Nanchang University, Nanchang, 330006 Jiangxi China

**Keywords:** Hepatocellular carcinoma, Hedgehog signaling pathway, FOXM1, TPX2, Proliferation

## Abstract

**Background:**

Aberrant activation of the Hedgehog (Hh) signaling pathway is frequently observed in hepatocellular carcinoma (HCC), nevertheless, the precise molecular mechanism remains unclear. Forkhead box M1 (FOXM1), a target of the Hh pathway, is a key oncofetal transcription factor and a master cell cycle regulator. Targeting protein for Xenopus kinesin-like protein 2 (TPX2) is an oncogene critical for mitosis. However, how these molecular events affect HCC progression remains unclear.

**Methods:**

Realtime PCR, immunohistochemistry, western blotting, and analyses of datasets TCGA and Gene Expression Omnibus (GEO) were conducted to assess the expression of TPX2 and FOXM1 at the mRNA and protein levels in HCC samples or HCC cells. Expression and knockdown of TPX2 and FOXM1 were performed to assess their role in regulating HCC cell proliferation in vitro and in vivo. Dual luciferase report assay and chromosome immunoprecipitation (ChIP) were investigated to seek the FOXM1 binding sites in the promoter of TPX2.

**Results:**

Specific antagonists (cyclopamine and GANT61) of the Hh pathway down-regulated TPX2, whereas activation of Hh signaling stimulated TPX2 expression. Furthermore, TPX2 over-expression accelerated HCC cell proliferation when upstream events of Hh signaling were inhibited, and TPX2 knockdown significantly alleviated Sonic Hh ligand (Shh)-induced HCC cell proliferation. Reporter assays and ChIP showed that FOXM1 bound to the TPX2 promoter, confirming that TPX2 is a direct downstream target of FOXM1. Xenograft model further verified the cell function and expression regulation of TPX2 and FOXM1 in vivo. Furthermore, FOXM1 regulated TPX2 activity to drive HCC proliferation. Immunohistochemical (IHC) analysis indicated that FOXM1 and TPX2 were highly-expressed in HCC samples and cohort study revealed that FOXM1 and TPX2 may act as negative predictors for the prognosis of patients with HCC.

**Conclusions:**

TPX2 acts as a novel downstream target and effector of the Hh pathway, and Hh signaling contributes to HCC proliferation via regulating the FOXM1-TPX2 cascade, suggesting that this signaling axis may be a novel therapeutic target for HCC.

**Graphical abstract:**

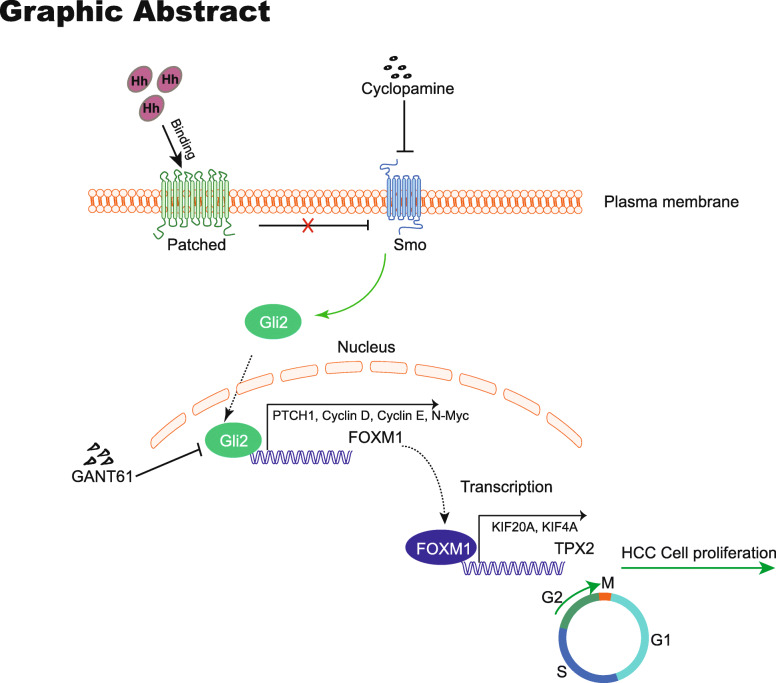

## Background

Liver cancer is the sixth commonly diagnosed cancer and the fourth leading cause of cancer-related mortality worldwide [1]. HCC, which is derived from hepatocytes, accounts for 70–90% of primary liver cancers in most parts of the world [2]. Current therapeutic regimens offer little improvement on this dismal prognosis of HCC, as patients inevitably show relapse despite aggressive therapy. Therefore, identification of molecular mediators that endow HCC cells with sustainable proliferative capabilities are urgently required for predicting the risks of cancer progression and prognosis and developing effective target-specific therapy for patients with HCC.

Among the signaling cascades dysregulated in HCC, the canonical Hh signaling pathway plays a substantial role. In vertebrate species, Hh signaling pathway is initiated by the binding of the Sonic Hh (Shh) ligand to the receptor Patched-1 (PTCH1) on the cell membrane, leading to derepression of the signal transducer protein and proto-oncogene Smoothened (SMO), which then relays the Hh signaling activity to activate the terminal effectors, namely GLI1, GLI2, and GLI3 [3]. GLI1 consists of zinc finger domains and a C-terminal activator domain, and acts as a constitutive transcriptional activator, whereas GLI2 harbors both positive and negative regulatory domains, however, GLI3 plays the role of a transcriptional repressor [3]. In particular, deletion of GLI2 is embryonically lethal, whereas GLI1 is dispensable for mouse development or viability [4]. GLI2, instead of GLI1, is essential for the initial and ectopic activation of the Hh pathway [5]. GLI2 regulates the transcription of not only genes directly associated with cellular proliferation, such as MYC, CCND1/2, CCNB1, and CCNE [6], but also those encoding transcription factors, such as FOXM1 [6, 7], which further activates more target genes. Despite that, it remains unclear whether there are other new target genes of Hh pathway and their roles in cell proliferation.

FOXM1, a Forkhead Box (Fox) protein, is not expressed in terminally differentiated, non-dividing cells whereas it is specifically active in proliferating epithelial and mesenchymal cells [8], where it activates cyclins of both G1/S progression and G2/M transition to promote cell cycle progression [9]. Cultured hepatocytes over-expressing FOXM1 were more efficient than control cells in repopulating injured mice liver. Furthermore, mice lacking FOXM1 in hepatocytes displayed severely impaired regenerative capacity after partial hepatectomy [10]. Therefore, FOXM1 is critical for normal functioning as it facilitates cell proliferation. Nevertheless, when aberrantly activated, it functions as an oncogene in human cancers such as HCC [11]. Mouse hepatocytes with conditional deletion of FOXM1 significantly resisted to the development of diethylnitrosamine/phenobarbital-induced HCC [12]. Hence, FOXM1 is a promising target for cancer therapy [13]. However, FOXM1 signaling is complex owing to its cooperation with the Wnt [14] and TGF-β signaling pathways [15] and other factors [16], and hence the mechanisms underlying its involvement in HCC development remains to be elucidated.

TPX2 is another protein closely related to the cell cycle; it is best known for its critical roles in mitosis and spindle assembly, which is indispensable for normal cell division. TPX2 was initially reported to exist exclusively in the nuclei of proliferating cells, but changed its sub-nuclear localization with cell cycle progression [17] to appear diffused all over the nucleus in interphase. TPX2 is closely associated with the spindle pole and the mitotic spindle during mitosis, followed by rapid degradation after completion of cytokinesis. Upon entering the mitotic phase, RanGTP, induced by chromosomes in their vicinity, dissociates TPX2 from a protein complex containing importin α and β; the released TPX2 acts as a microtubule-associated protein in the early steps of microtubule nucleation to form spindle in the vicinity of the chromosomes. At metaphase, TPX2 accumulates at spindles poles in a dynein-dynactin-dependent manner and is essential for spindle pole integrity; it also targets other proteins, such as Xklp2, the kinesin-5 motor Eg5 [18], adducin-1 [19] and the kinase Aurora A [20] to the spindle. Aurora A is activated by TPX2 after being targeted to spindle, and in turn it phosphorylates TPX2 [20]. The coordination between TPX2 and its partners is required for constructing a correct spindle. Although TPX2 has been reported to be up-regulated in liver tumors [21], the precise mechanisms underlying its functions and regulation of its expression in HCC require further investigation.

To address the above questions, we performed a series of bioinformatic analyses and experiments to elect TPX2 as a putative target of Hh signaling. Our result discovered the critical role of Hh-FOXM1-TPX2 signaling in HCC proliferation. Our study also suggested that this pathway may be a potential target for HCC therapy.

## Materials and methods

### Small molecular reagents and constructs

The GLI inhibitor GANT61 (G9048), dimethyl sulfoxide (DMSO) (D2650), protease inhibitor cocktail (P8340), and polyethylenimine (PEI) transfection reagent (408727) were purchased from Sigma-Aldrich (St. Louis, MO, USA). DMSO was used as the solvent for some reagents and also as the vehicle control. The SMO inhibitor cyclopamine (S1146) was purchased from Sellect Chemicals (Houston, TX, USA). Puromycin (P8230) was purchased from Solarbio (Beijing, China). Lipofectamine 2000 transfection reagent (11668019) and TRIzol reagent (15596018) were from Thermo Fisher Scientific (Waltham, MA, USA). All other chemicals used were of analytical grade and were purchased from Sigma-Aldrich (St. Louis, MO).

The human full-length FOXM1 (NM_202002) construct was subcloned into the mammalian expression vector pcDNA3.1-Myc/His A (V855–20; Invitrogen, Carlsbad, CA, USA) using the In-Fusion cloning kit (639,619; Clontech Laboratories, Mountain View, CA, USA) to obtain the expression plasmid pcDNA3.1-Myc/HisA-FOXM1. The FOXM1 and TPX2 short hairpin (sh) RNA constructs were generated using the BLOCK-iT™ Pol II miR-RNAi expression vector kit (K4936–00; Invitrogen, Carlsbad, CA, USA) as described previously [22]. Knockdown efficiency was determined using reverse transcription-quantitative PCR (RT-qPCR) and western blot (WB) analysis. The oligo nucleotide sequences for the shRNA constructs are listed in **Supplementary Table S****1**. The authenticity of all constructs was verified using DNA sequencing.

### Cell lines and culture

HEK293T (RRID: CVCL_0063) and the HCC cell line HepG2 (RRID: CVCL_0027) were purchased from the American Type Culture Collection (ATCC, Manassas, VA, USA) in 2016. Huh7 (RRID: CVCL_0336) and Hep3B (RRID: CVCL_0326) from the National Infrastructure of Cell Line Resource (Beijing, China) in 2018. HCC-LM3 (RRID: CVCL_6832) was obtained from the Cell Bank of the Type Culture Collection of the Chinese Academy of Sciences (Shanghai, China) in 2016. All cell lines were authenticated using short tandem repeat profiling and were negative for mycoplasma contamination detecting via PCR-based assay (performed in February 2018). The used cells were immediately expanded and frozen so that they could be resuscitated every 3 to 4 months from a frozen vial of the same batch of cells. HEK293T, Huh7, HCC-LM3 and HepG2 were cultured in Dulbecco’s Modified Eagle’s Medium (DMEM) (C11995500BT; Gibco; Grand Island; NY; USA), and the Hep3B cells were cultured in Minimum Essential Medium (MEM) (C11095500BT; Gibco), all supplemented with 10% FBS (10091148; Gibco) and 1% penicillin/streptomycin (15140–122; Gibco) at 37 °C in a humidified 5% CO_2_ atmosphere. Cells were transiently transfected with Lipofectamine 2000 for HCC cells or with PEI for HEK293T cells according to the manufacturer’s instructions. In all experiments, the medium was replaced daily.

A short hairpin RNA lentivirus system targeting the 5′-AGCAAGTTGAAGACTTCCATA-3′ sequence of the TPX2 mRNA (hereafter referred to collectively as shTPX2–1684), 5′-GATGTTGTGGGTGTTCCTGAA-3′ sequence of the TPX2 mRNA (hereafter referred to collectively as shTPX2–2103), and the 5′-TGTCTCGGAAATGCTTGTGAT-3′ sequence of the FOXM1 mRNA (hereafter referred to collectively as shFOXM1) was produced using the psi-LVRU6MP vector by GeneCopoeia (Guangzhou, China). All lentivirus infections were performed according to the manufacturer’s instructions. Stably infected cells were selected using puromycin (Hep3B: 0.5 μg/ml, Huh7: 2 μg/ml, and HepG2: 1 μg/ml) for 7 days. Knockdown efficiency was determined using WB analysis and RT-qPCR.

### RNA isolation, cDNA synthesis, and RT-qPCR

Total RNAs were extracted from fresh cells using the TRIzol reagent. One microgram total RNA was reverse-transcribed and cDNAs were synthesized using the PrimeScript RT reagent kit (RR047A; Takara Bio, Otsu, Japan) according to the manufacturer’s instructions. RT-qPCR was performed in triplicate using the SYBR Premix Ex Taq RT-PCR kit (RR820A; Takara Bio, Japan), and was analyzed using the Applied Biosystems StepOne Plus™ real-time PCR detection system (ABI, Foster City, CA, USA). The mRNA expression levels of the target genes were normalized to that of GAPDH and quantified with the ΔΔCT method. The specific primer sequences for PCR amplification are listed in **Supplementary Table S****2**. Each experiment was repeated at least thrice to obtain consistent results. Data were showed as means ± standard deviation.

### Hh target gene screening

Huh7 and HepG2 cells were treated with GANT61 (20 μM) or vehicle (DMSO) for 36 h, and cells were then harvested for RNA extraction. Gene expression profiles were determined via next generation sequencing (NGS) with Illumina NovaSeq by Novogene Co. Ltd. (Beijing, China), and genes with expression changed over 2 folds were considered as differentially expressed genes (DEGs). All raw data is available at sequence read archive (SRA) with accession no. PRJNA592618. The function annotation of these DEGs were analysed within WebGestalt (WEB-based GEne SeT AnaLysis Toolkit, http://www.webgestalt.org/).

HCC-LM3 cells were treated with cyclopamine (20 μM), GANT61 (20 μM) or vehicle (DMSO) for 48 h. Gene expression profiles were obtained using the genome-wide HumanHT-12 v4 Expression BeadChip arrays (Illumina, San Diego, CA). Genes with a *DiffScore* less than − 50 or more than 50 were considered differentially expressed genes (DEGs).

### Western blot analysis and antibodies

Total protein extracts were harvested and subjected to WB as described previously [23]. Primary antibodies against the following proteins were used for WB: GLI2 (ab26056; Abcam), PTCH1 (ab55629; Abcam), FOXM1 (sc-376,471; Santa Cruz Biotechnology, CA, USA), TPX2 (12,245; Cell Signaling Technology), and glyceraldehyde 3-phosphate dehydrogenase (GAPDH) (MAB374; Millipore, Billerica, MA, USA). This was followed by incubation with horseradish peroxidase (HRP)-conjugated secondary antibodies, namely normal goat anti-mouse IgG (31,430; Thermo Scientific Pierce) or normal goat anti-rabbit IgG (31,460; Thermo Scientific Pierce), and the membranes were probed with SuperSignal™ West Femto Maximum Sensitivity Substrate ECL (34,095; Thermo Fisher Scientific Inc). The immunoblot films were digitalized with Epson V700 scanner, and intensity of major bands were quantitated using Image J (National Institutes of Health, Bethesda, MD, USA). Each experiment was repeated at least thrice.

### Cell proliferation assays

For the cell proliferation assays, lentivirus-infected HCC cells were seeded in 96-well plates at a density of 6000 cells per well. After 24 h, the culture medium was replaced by 50 μm EdU (5-ethynyl-2′-deoxyridine) solution diluted in fresh cell culture medium, and the cells were incubated for another 1–4 h. The cell-light EdU experiments were performed following the manufacturer’s instructions using Cell-Light™ EdU Apollo 488 (C10310–3) and 567 (C10310–1) In Vitro Kit (Guangzhou RiboBio Co., Ltd., China). Three biological repeats (*n* = 3) in different wells were investigated for each treatment, and three fields of cells were counted each well, while only one representative field of each treatment was shown. Images were captured using an inverted fluorescence microscope (IX71; Olympus, Tokyo, Japan) at the same time and under the same experimental conditions, and data was analysed using the ImageJ.

For obtaining cell growth curve, lentivirus-infected HCC cells were seeded in 24-well plates at a density of 5000 cells per well in triplicate, and the cells were counted using flow cytometry every 24 h for consecutive 7 days after plating. The final data were displayed as fold increase relative to the cell numbers on day 1. For the clonogenic assay, lentivirus-infected HCC cells (2000–8000 cells per well) were seeded in 6-well plates and cultured for approximately 2 weeks. The culture media was changed every 2 days. The colonies were fixed with 4% paraformaldehyde and stained with 0.5% (w/v) crystal violet. Then, the plates were left to dry at room temperature. The Epson V700 scanner was used to scan and acquire a clear image, which was quantified using the ImageJ software.

The soft agar colony formation assay was performed to investigate the role of TPX2 in anchorage-independent cell growth as mentioned below. The lentivirus-infected HCC cells were seeded in 6-well plates in growth medium containing 0.7% agar (1 ml per well) on top of a layer of growth medium containing 1.2% agar (1.4 ml per well). A 500-μl mixture of growth medium with 10% FBS was added to the top of the agar, which was replenished every 3 days. The cell suspension was plated and cultured for approximately 20 days. Colonies larger than 50 μm were imaged and counted for the assay. One representative field for each filter is shown in the figures.

### Flow cytometry analysis

For DNA content analysis, cells were fixed in ice-cold 70% methanol for 2 h at − 20 °C, washed, rehydrated, and resuspended in phosphate buffered saline (PBS). Finally, 50 μg/ml propidium iodide (PI) (P4864; Sigma-Aldrich) and 100 μg/ml RNase A (EN0531; Thermo Fisher Scientific) were added and incubated for 30 min at 4 °C. The samples were analysed using the Accuri C6 Plus flow cytometer (BD Biosciences, San Jose, CA, USA). Data were analyzed with Modfit software (Verity, Topsham, ME, USA).

### Dual luciferase assay

The full-length luciferase reporter constructs and its variants used for detection of TPX2 transcriptional activation by FOXM1 were constructed by inserting the TPX2 promoter sequence into the pGL4.20 firefly luciferase vector (E6751; Promega, Madison, WI, USA), followed by deletion of some specific fragments using a mutagenesis kit (SMK-101; Toyobo, Osaka, Japan). In addition, FOXM1 binding sites 9, 10, and 11 in pGL4.20-TPX2- 2B were mutated to validate their roles in FOXM1-mediated TPX2 activation. All primers for the luciferase reporter constructs are shown in **Supplementary Table S****3**. The cloned promoter sequences were validated using DNA sequencing.

HepG2 cells that were 70% confluent in 24-well plates were transfected in triplicate with 0.4 μg pGL4.20-TPX2 promoter-luciferase reporters and 0.15 μg FOXM1-expressing plasmid or empty vector along with 0.015 μg pRL-TK for normalization. After 48 h, luciferase activity was determined using a luminometer and the dual-luciferase reporter system (E1910; Promega) following the manufacturer’s instructions. The activity of the pGL4.20-TPX2 promoter-luciferase reporter was normalized to that of the pRL-TK Rluc reporter, and was compared between HepG2 cells transfected with the FOXM1 expression plasmid or empty vector. The above experiments were repeated at least thrice.

### Chromatin immunoprecipitation (ChIP)

Briefly, Huh7 cells were grown to 90% confluence and were cross-linked with 1% (v/v) formaldehyde in PBS, followed by ChIP as described previously [24]. The fragments were mixed with an FOXM1 antibody (sc-376,471; Santa Cruz Biotechnology) and protein A-agarose beads (11,134,515,001; Roche, Palo Alto, CA, USA) to enrich DNA fragments bound to FOXM1 via immunoprecipitation. Normal mouse immunoglobulin (IgG) (sc-2025; Santa Cruz Biotechnology) was used as a control for the ChIP assay. The ChIP PCR primer sequences are listed in **Supplementary Table S****4**. The experiment was repeated at least thrice.

### Subcutaneous xenograft assay

For in vivo experiments, 2 × 10^7^ Huh7 cells stably expressing sh-Control or sh-FOXM1 were digested by trypsin, following by resuspended in sterile PBS (200 μL) and then were injected subcutaneously into the flanks of 5-week-old female BALB/c-nu athymic nude mice (SLAC Laboratory Animal CO. Ltd., Hunan, China; 5 mice per group). Subcutaneous tumor formation was observed starting 7 days post-injection, the mice were administered 2 μg/mL doxycycline and 5% sucrose in sterile drinking water. And tumor sizes were measured thrice weekly using Vernier calipers. Tumor volume was calculated with the formula: (length × width^2^)/2. At 24 days after injection, tumors were harvested for immunohistochemistry and western blotting. Protocols for animal experiments were approved by the Ethical Committee of the First Affiliated Hospital of Nanchang University and conformed to the guidelines of the National Institutes of Health on the ethical use of animals.

All surgeries were performed under sodium pentobarbital anesthesia, with minimized suffering.

### Patients and clinical tissue samples

A cohort of 68 patients diagnosed with primary HCC who underwent surgical resection at the First Affiliated Hospital of Nanchang University between January 2010 and June 2018 (age > 18 years) were included in the current study. All patients were diagnosed based on the histopathological criteria, and none had received chemotherapy, radiotherapy, or immunotherapy before surgery. The specimens were reviewed by a pathologist to ensure that the samples included both tumors and adjacent non-malignant tissues. The histological classifications were defined according to the World Health Organization criteria as grade I (*n* = 19), grade II (*n* = 34), and grade III (*n* = 13). HCC clinical stages were defined according to the American Joint Committee on Cancer (AJCC, the 8th edition, 2010). All relevant ethical regulations were followed. Clinical samples were collected after an informed written consent was obtained from the participants in accordance with the Ethics Committee requirements at the participating institutes and the World Medical Association’s Declaration of Helsinki. All experiments with human tissue samples were approved by the Ethics Committee of the First Affiliated Hospital of Nanchang University (Nanchang, China). Detailed clinical and pathological information has been summarized in **Table 1**.

### Histopathological analysis and immunohistochemistry (IHC)

The excised clinical samples were fixed in a 10% neutral buffered formalin solution, dehydrated and embedded into paraffin wax blocks. Embedded-tissues from human HCC samples were cut into 3-μm-thick sections, mounted onto slides, and processed for histopathological evaluation. All samples were stained with hematoxylin and eosin (H&E) and immunohistochemistry procedures were performed as described previously [23]. Briefly, the tissues were de-paraffinized, rehydrated, and treated with 3% hydrogen peroxide to block endogenous peroxidase activity. Then, the tissues were treated with EDTA (pH 9.0) and heated in a microwave for 45 min. Following a standard antigen retrieval protocol, the slides were incubated with the appropriate primary antibody (FOXM1 (sc-502; Santa Cruz Biotechnology), TPX2 (12,245; Cell Signaling Technology) overnight at 4 °C in a humidified chamber. Subsequently, the slides were rinsed with PBS and incubated for 30 min at 37 °C with appropriate biotinylated immunoglobulins (PV-6000; Zhongshan Biotechnology, China). Then a Polink-2 HRP DAB detection kit (ZLI-9018; Zhongshan Biotechnology, China) was used to visualize the immunoreactivity. A negative control was set up in each case with normal IgG. IHC images were captured using a FSX100 microscope equipped with a digital camera system (Olympus). The German semi-quantitative scoring system was used to evaluate the staining intensity and area of the target genes. The intensity of nuclear staining was evaluated and scored independently by three qualified pathologists, and graded semi-quantitatively as described previously [7]. The final scores, which ranged from 0 to 12, were derived by multiplying the percentage and intensity scores. Patients were divided into two groups according to their immunohistochemistry scores compared to medium value, namely those with scores of 0 to 6 were considered ‘Low expression’ and those with scores of 6 to 12 as ‘High expression’.

### Analysis of patient survival and FOXM1 and TPX2 expression levels in tumor samples

For Kaplan-Meier Plotter database analysis (http://kmplot.com/), the mRNA levels were normalized to the median value of the dataset and log2-transformed. Kaplan-Meier survival curves were plotted for patients with HCC, follow-up data of whom were available [25]. The Kaplan-Meier method and log-rank (Mantel-Cox) test was applied to analyze survival differences between groups.

### Statistical analysis

Data were presented as the means ± SD of independent experiments performed at least thrice. Paired or unpaired two-tailed Student’s *t*-test was used to assess the statistical significance of differences between two different groups of quantitative data. One-way analysis of variance (ANOVA) was used to compare the continuous cell or tumor volume growth curve between two groups. Correlations between target gene expression and clinicopathological characteristics were analyzed using χ^2^ tests, and statistical significance was analyzed using Fisher’s exact test for qualitative comparison. Comparison of IHC scores between two different groups, for example, in carcinoma and adjacent tissue group, was performed using the Mann Whitney *U* test. Correlation analysis of IHC scores for FOXM1 and TPX2 expression was performed using Pearson’s Chi-squared test. Correlation was defined as follows: strong (*r*^*2*^>0.75), good (0.4 ≤ *r*^*2*^ ≤ 0.75), and poor (*r*^*2*^ < 0.4). *p* < 0.05 (*) and *p* < 0.01 (**) indicated statistically significant changes. The SPSS software version 21.0 (SPSS, Chicago, IL, USA) was used for data analyses.

## Results

### TPX2 expression was regulated by the Hh signaling pathway

**Fig. 1:**
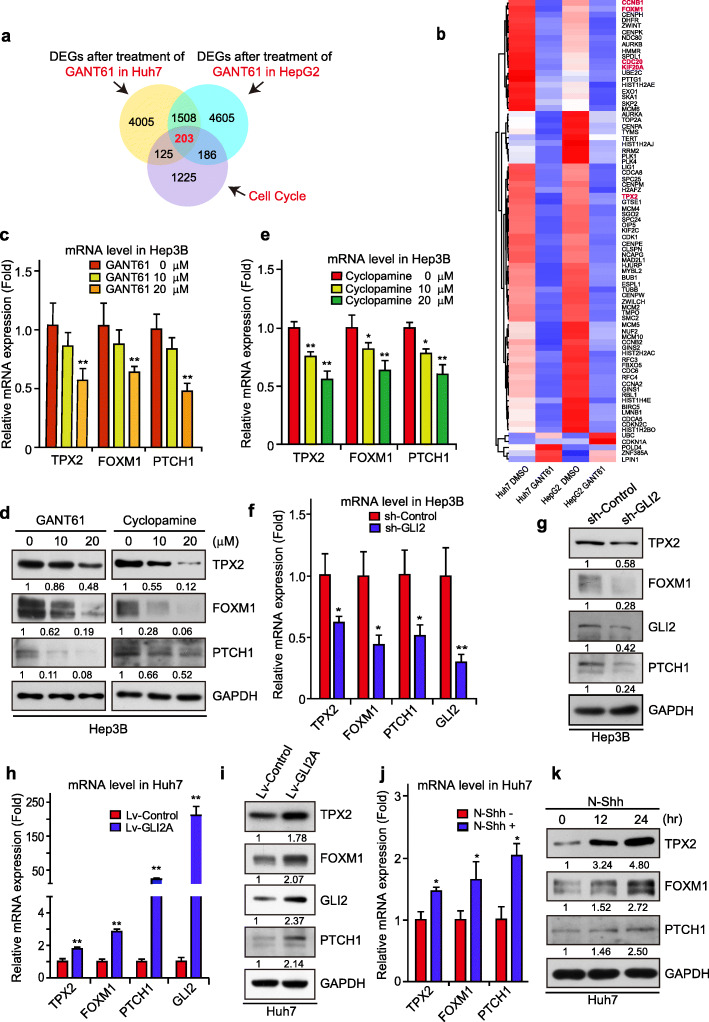
TPX2 expression is regulated by the Hh signaling pathway. **a.** Venn diagrams of differentially expressed genes (DEGs) in Huh7 and HepG2 cells after treating with GANT61 versus genes enriched in “Cell Cycle” gene set. **b.** Representative candidate genes derived from Venn diagrams in Fig. 1a were represented in a heat map. Red signal denotes higher expression and blue signal denotes lower expression. Gene names marked in red are previously reported genes regulated by FOXM1. **c.** Hep3B cells were treated with GANT61 (10 ~ 20 μM) for 48 h and harvested for real-time PCR analysis with the indicated primers. **d.** Hep3B cells were treated with GANT61 (left panel) or cyclopamine (right panel) (10 ~ 20 μM) for 48 h and harvested for WB analysis with the indicated antibodies. **e.** Hep3B cells were treated with cyclopamine (10 ~ 20 μM) for 48 h and harvested for real-time PCR analysis with the indicated primers. **f-g.** Hep3B cells transfected with shRNA-control or shRNA-GLI2 was harvested for real-time PCR analysis with the indicated primers (**f**) and for WB analysis with the indicated antibodies (**g**). **h-i.** Huh7cells transfected with Lv-control or Lv-GLI2A were subjected to real-time PCR analysis with the indicated primers (**h**) as well as WB analysis with the indicated antibodies (**i**). **j.** Huh7 cells incubated with or without N-Shh were subjected to real-time PCR analysis with the indicated primers. **k.** Huh7 cells incubated with N-Shh for 0, 12, and 24 h were harvested for WB analysis with the indicated antibodies. Data was shown as mean ± SD (*n* = 3). *, *p* < 0.05; **, *p* < 0.01

To further investigate the effects of aberrant Hh signaling activation on the tumorigenesis or development of HCC, gene expression profiles of HCC cells were determined by RNA-Seq after GANT61, an antagonist of Gli transcriptional factors [26], treatment. As shown in Fig. 1a, 1711 genes response to Hh attenuation in both Huh7 and HepG2 cells by GANT61, which were considered as DEGs. The function annotation of these DEGs revealed that Hh signaling might affect the cell cycle and its regulatory process in HCC cells (Fig. S1a), thus we further overlapped the down-regulated genes with genes related with cell cycle (GO:0007049), and a Venn cluster analysis was conducted, which discovered 203 of the down-regulated genes were relevant to cell cycle (Fig. 1a). Among these 203 genes, many had been reported as GLI target genes involved in cell proliferation, such as KIF20A, FOXM1, and CCNB1 (Fig. 1b), which may act as positive controls for confirming the authenticity of our screening results. And TPX2, which was substantially down-regulated in both Huh7 and HepG2 by GANT61 (Fig. 1b), was an interesting candidate for further analysis because of its critical role in spindle formation and maintenance [27–29], which is indispensable for normal cell division and proliferation. Therefore, we validated the RNA-Seq screening by qPCR, which confirmed that GANT61 reduces TPX2 expression in both Huh7 (Fig. S1b) and HepG2 (Fig. S1c) cells. Besides, in our previous experiments screening via microarray, TPX2 was also identified as Hh regulated gene (Fig. S1d-e), and the regulation were also validated by qPCR (Fig. S1f-g).

To further confirm the regulation of TPX2 in HCC cells, the protein level of TPX2, together with its mRNA level, were determined in Hep3B, another HCC cell line, and results revealed that GANT61 would decrease both the mRNA level (Fig. 1c) and protein level (Fig. 1d) of TPX2 in Hep3B cells. Moreover, cyclopamine, an canonical Hh signaling inhibitor targeting SMO, repressed the expression of TPX2 in protein level and mRNA level in multiple HCC cells, as well (Fig. 1d-e, and S1h-i), which further indicates that pharmacological attenuation of Hh signaling decreases the expression of TPX2.

Gli proteins are the most important transcriptional factors mediating Hh signaling from cytoplasm into nucleus. Blocking Hh signaling via knockdown Gli2 led to the depression of TPX2 expression, together with other canonical Hh target genes (Fig. 1f-g), while activating Hh signaling via ectopic expression of activated Gli2 (Gli2A) increased TPX2 level (Fig. 1h-i). We then activates Hh signaling by Hh ligand, N-Shh, and it revealed that N-Shh stimulation up-regulates the expression of TPX2 (Fig. 1j-k), offering more evidence indicating that TPX2 is a target gene downstream Hh signaling.

Taken together, these results indicated a critical role of Hh/GLI signaling in regulating TPX2 transcription.

### TPX2 over-expression promoted HCC proliferation despite inhibition of upstream Hh/GLI signaling

**Fig. 2:**
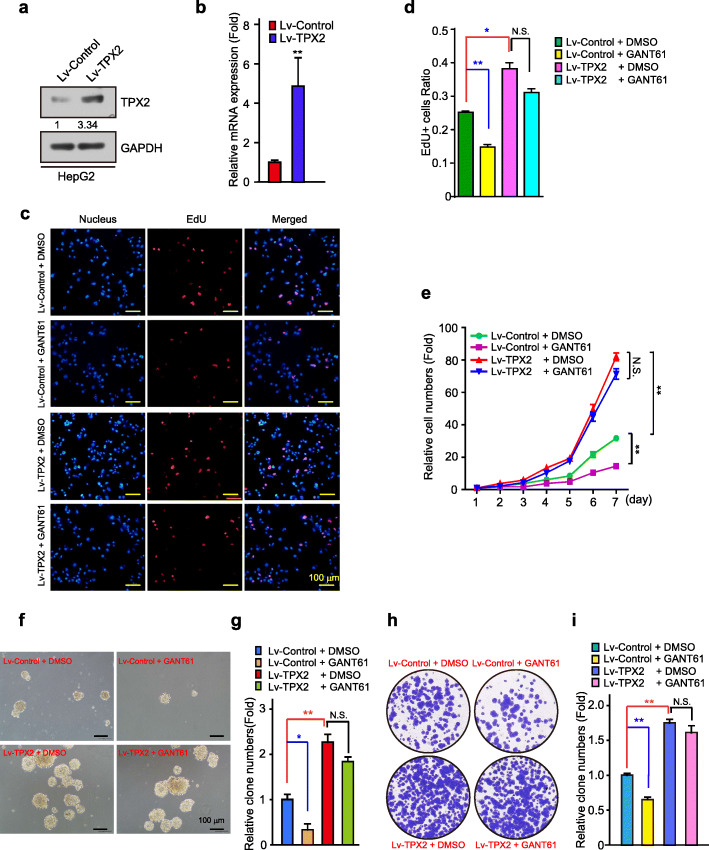
Over-expression of TPX2 promotes HCC proliferation despite inhibition of Hh/GLI signaling. **a-b.** Validation of HepG2 cells stably over-expressing TPX2 using WB analysis with the indicated antibodies (**a**) and real-time PCR with the indicated primers (**b**). **c.** Comparison of the proliferative ability of Lv-control + DMSO, Lv-control + GANT61 (2 μM), Lv-TPX2 + DMSO, and Lv-TPX2 + GANT61 (2 μM) in HepG2 cells treated with EdU. Scale bar, 100 μm. **d.** The ratio of EdU-positive cells was quantified using the ImageJ software (*n* = 3). **e.** Cell growth curves of Lv-control + DMSO, Lv-control + GANT61 (2 μM), Lv-TPX2 + DMSO, and Lv-TPX2 + GANT61 (2 μM) in HepG2 cells. **f-i.** Comparison of the proliferative ability of Lv-control + DMSO, Lv-control + GANT61 (2 μM), Lv-TPX2 + DMSO, and Lv-TPX2 + GANT61 (2 μM) in HepG2 cells using soft-agar colony formation assays (**f**) and plate colony formation assay (**h**). Soft-agar colonies (**g**) and plate colonies (**i**) were counted using the ImageJ software. Scale bar, 100 μm. Data was shown as mean ± SD (*n =* 3). *, *p* < 0.05; **, *p* < 0.01, N.S. denotes not significant

Several high profile cases have identified TPX2 as an oncogene that promotes cancer proliferation and tumorigenesis [21, 30, 31]. To elucidate the biological effect of TPX2 on Hh/GLI signaling-dependent growth of HCC cells, HepG2 and SK-Hep-1 cells, in which the expression level of TPX2 was relatively lower (Fig. S7d), were infected by lentivirus to generate cell lines with TPX2 stably expressing (Fig. 2a-b and S2a). And multiple assays were conducted to testify the effect of TPX2 and Hh signaling on cell proliferation, including EdU staining, cell growth curve, and colony formation assay. In these assays, EdU staining, which represents the DNA synthesis in cells, directly demonstrates the cell proliferation state of cells, and cell growth curve and colony formation reveal the final results of the cell proliferation and growth.

In accordance with other studies, GANT61 reduced the ratio of EdU-positive cells (Fig. 2c-d and S2b-c), suppressed cell growth (Fig. 2e and S2d) and colony formation (Fig. 2f-I and S2e-f) in both HepG2 and SK-Hep-1 cells, while ectopic expression of TPX2 accelerated cell growth (Fig. 2c-I and S2b-f). Interestingly, with TPX2 expression, the inhibitory effect of GANT61 were almost eliminated (Fig. 2c-I and S2b-f), which indicates that Hh signaling regulates cell proliferation and cell growth through a TPX2 dependent pathway.

In conclusion, these results indicated that TPX2 may significantly rescue the GANT61-induced proliferation phenotypes of HCC cells, and that TPX2 is a downstream effector of Hh/GLI signaling-dependent HCC proliferation.

### TPX2 depletion inhibited Hh/GLI signaling-induced HCC cell proliferation

**Fig. 3:**
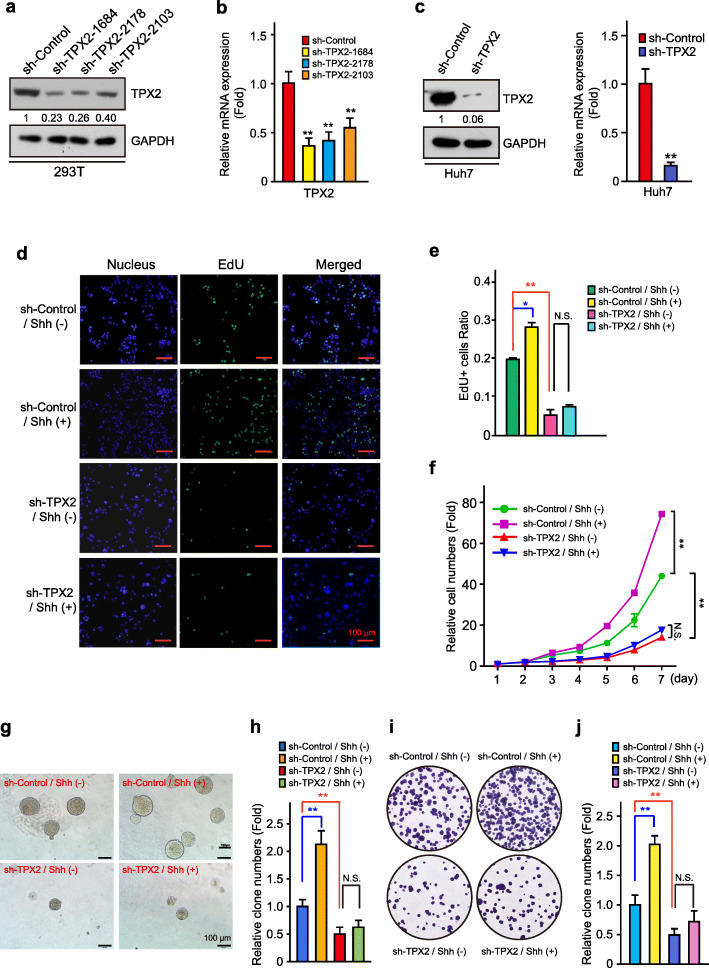
TPX2 abrogation inhibits Hh signaling-induced HCC cell proliferation. **a-b.** HEK293 cells were transfected with shRNA-Control or three shRNA-TPX2 constructs for 48 h and were harvested for WB analysis with the indicated antibodies (**a**) and real-time PCR analysis with the indicated primers (**b**). **c.** The most efficient shRNA sequence for knocking down TPX2 was selected to construct lentivirus systems. Validation of Huh7 sh-TPX2 stable cell lines using WB analysis (left panel) and quantitative real-time PCR (right panel). **d.** TPX2 abrogation inhibited Hh signaling-induced HCC cell proliferation as determined using EdU staining. Scale bar, 100 μm. **e.** The ratio of EdU-positive cells was quantified using the ImageJ software (*n =* 3). **f.** Cell growth curves of sh-Control / Shh (−), sh-Control / Shh (+), sh-TPX2 / Shh (−), and sh-TPX2 / Shh (+) in Huh7 cells. **g-j.** Comparison of the proliferative ability of sh-Control / Shh(−), sh-Control / Shh (+), sh-TPX2 / Shh(−), and sh-TPX2 / Shh (+) in Huh7 cells using soft-agar colony formation assays (**g**) and plate colony formation assay (**i**). Soft-agar colonies (**h**) and plate colonies (**j**) were counted using the ImageJ software. Scale bar, 100 μm. Data was shown as mean ± SD (*n =* 3). *, *p* < 0.05; **, *p* < 0.01, N.S. denotes not significant

Next, we investigated the effects of TPX2 knockdown on Hh/GLI signaling-dependent HCC proliferation. Three shRNAs were designed and constructed (Fig. 3a-b), and Huh7 and Hep3B, in which expression level of TPX2 were relatively higher (Fig. S7d), were then infected by lentiviruses containing these shRNAs to generated stable cell lines with TPX2 knockdown (Fig. 3c, S3a and S4a). Consistent with previous study, TPX2 depletion reduced the protein level of PCNA (Fig. S3a), a widely-used marker gene of cell proliferation, which demonstrated the proto-oncogene properties of TPX2 in HCC cells. Similar to previous study, EdU staining, cell growth curve, and colony formation assay were conducted in these stable cell lines to further testify the proto-oncogene properties of TPX2 and Hh signaling.

As expected, TPX2 knockdown decreased the proportion of EdU-positive cells (Fig. 3d-e, S3b-c and S4b-c), retarded cell growth (Fig. 3f and S3d) and colony formation rates (Fig. 3g-j and S3e-f), while Hh signaling activated via N-Shh conditional medium treatment showed a promotion in cell proliferation and cell growth in these assays (Fig. 3d-j, S3b-f and S4b-c). Furthermore, the depletion of TPX2 blocked the regulatory effect of Hh signaling on cell proliferation in Huh-7 cells (Fig. 3d-j and S4b-c) or dramatically restrained its function in Hep3B cells (S3b-g), which further indicates that Hh signaling might expedite cell proliferation and cell growth via regulating TPX2.

In summary, these results demonstrated that TPX2 depletion can significantly alleviate Hh/GLI signaling activation-mediated HCC cell proliferation.

### TPX2 was a direct target of FOXM1

**Fig. 4:**
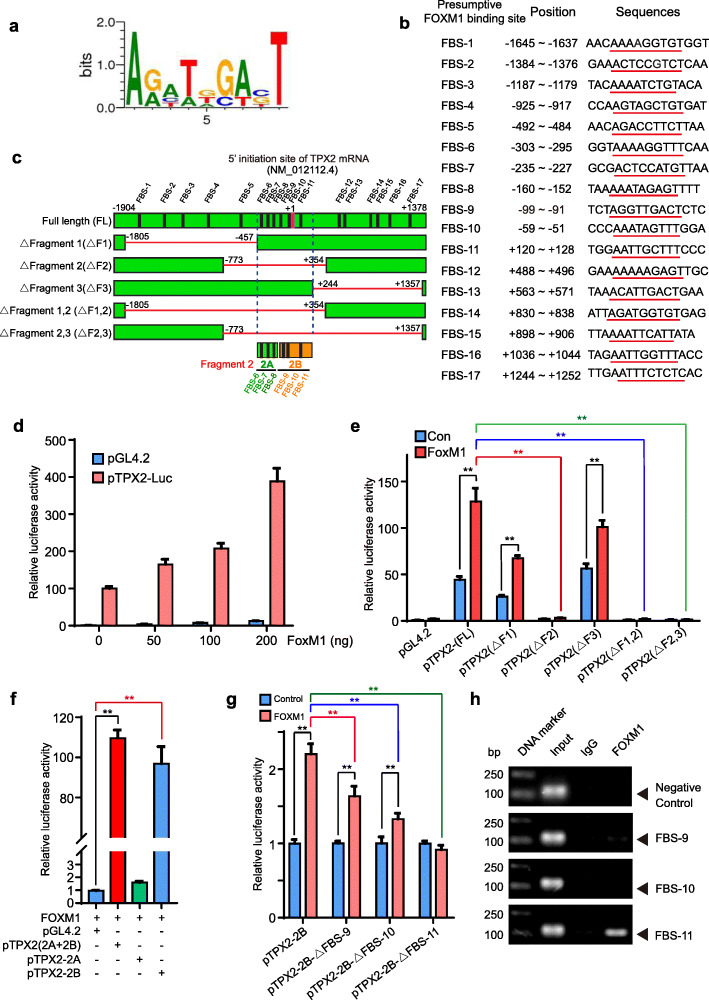
FOXM1 directly activates TPX2 transcription. **a.** Consensus sequence of FOXM1-binding site, sourced from MotifMap [33]. **b.** Predicted FOXM1-binding sites within the genomic sequence adjacent to the transcription start site of TPX2. The underlined sequences with red lines are core sequence of FOXM1-binding sites. **c.** Schematic diagram showing candidate FOXM1-binding site within the TPX2 promoter, the full-length luciferase reporter construct, and its deletion variants with different FOXM1-binding sites. **d.** Dual luciferase assay of TPX2 promoter-luciferase reporters in FOXM1-expressing or control HepG2 cells. **e-g.** Dual luciferase assay of sets of TPX2 promoter truncation-luciferase reporters in FOXM1-expressing or control HepG2 cells. **h.** ChIP assay in Huh7 cells to detect FOXM1 binding to the TPX2 promoter. Data was represented as mean ± SD of three independent experiments. **, *p* < 0.01

The above observations suggested that Hh signaling may facilitate TPX2 expression to mediate its function in cell proliferation. However, among the predicted transcription factor binding sites identified using the online program MatInspector professional version 7.2 from Genomatics (www.genomatix.de/) [32], were multiple potential FOXM1-binding sites (FBS: 5′-TAAACA-3′) within the − 2000 ~ + 1500 genomic region (the 5′ initiation site of TPX2 [NM_012112.4] was numbered as + 1) instead of the conserved GLI-binding site (Fig. 4a-b). So, we reasoned that TPX2 is likely a direct target of FOXM1, indicating that TPX2 transcription can be directly activated by FOXM1 in response to GLI2 [34]. For validating this hypothesis, genomic sequences containing all the 17 putative FBS were cloned into the pGL4.20 plasmid vector for the dual-luciferase assay, yielding six luciferase reporter constructs, namely Full Length (FL), delta-Fragment 1 (△F1), delta-Fragment 2 (△F2), delta-Fragment 3 (△F3), delta-Fragment 1, 2 (△F1, 2), delta-Fragment 2, 3 (△F2, 3) (Fig. 4c).

The constructs, along with pRL-TK for normalization, were co-transfected in HepG2 cells. The cells were harvested 48 h after the transfection, the luciferase activity was measured, and the exogenous expression of FOXM1 were testified via immunoblotting. Results of the reporter gene assays showed that over-expressed FOXM1 activated the transcription of the TPX2 promoter in a dose-dependent manner (Fig. 4d and S5a), indicating that TPX2 was specifically activated by FOXM1. Subsequently, we observed that deletion of a fragment containing FBS-6, 7, 8, 9, 10, and 11 completely abrogated the activation of the TPX2-Luci reporter by FOXM1 (Fig. 4e and S5b). This fragment, named Fragment 2, was further divided into Fragment 2A containing FBS-6, 7, and 8 and Fragment 2B containing FBS-9, 10, and 11 to precisely define the FOXM1-responsive region within the TPX2 promoter (Fig. 4f and S5c). Results showed that the response of Fragment 2B to FOXM1 was comparable to that of Fragment 2, while Fragment 2A displayed almost no response to FOXM1 (Fig. 4f). When FBS 9, 10, and 11 were each deleted from Fragment 2B, the TPX2-Luci reporter activity in response to FOXM1 was significantly suppressed, with deletion of FBS-11 completely suppressing the activity (Fig. 4g and S5d), indicating that these three sites were involved in activation of TPX2 by FOXM1. This was further supported by the results of ChIP assay, where compared to IgG, the anti-FOXM1 antibody specifically enriched the genomic sequences containing FBS-11 (Fig. 4h), strongly suggesting that FOXM1 binds to the TPX2 promoter. Thus, TPX2 is a novel target of FOXM1.

### TPX2 expression was regulated by Hh-FOXM1 signaling

**Fig. 5:**
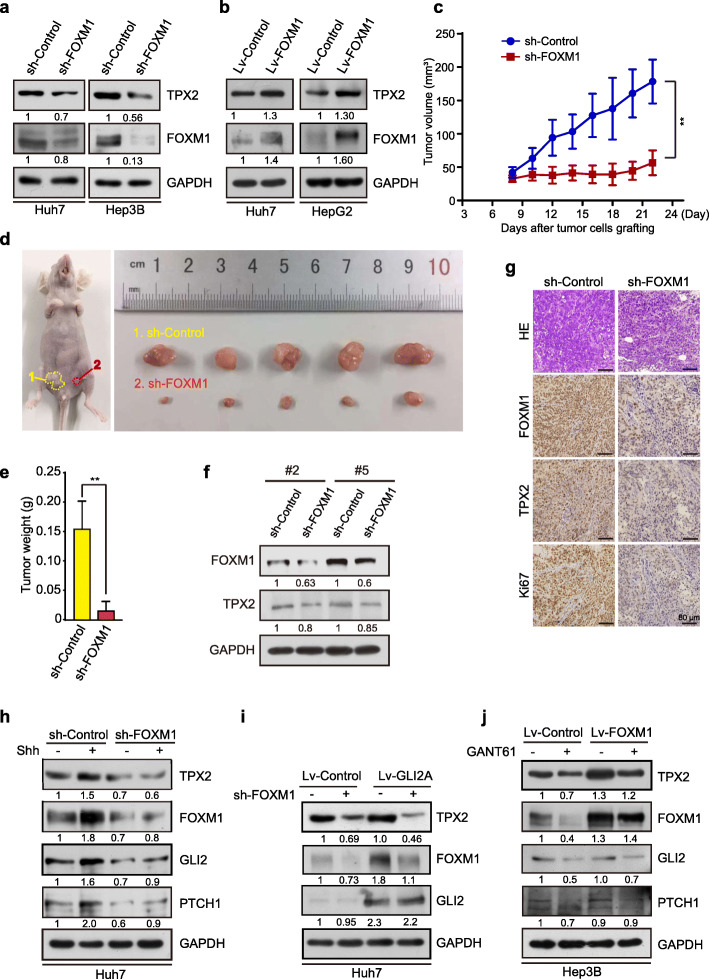
TPX2 expression is regulated by Hh-FOXM1 signaling. **a.** Protein expression levels of TPX2 and FOXM1 were assessed after stable knock down of FOXM1 in Huh7 and Hep3B cells using WB analysis. **b.** WB analysis to determine protein expression levels of TPX2 and FOXM1 in Huh7 and HepG2 cells stably over-expressing FOXM1. **c-e.** FOXM1 depletion suppresses tumor growth in vivo. Huh7 cell lines (2 × 10^7^ cells) that expressing sh-FOXM1 or sh-Con were subcutaneously injected into nude mice on each side of the inguinal region. Mice were administered 2 μg/mL doxycycline and 5% sucrose in sterile drinking water. Xenografts were harvested after 3 weeks. Tumor sizes on either side were monitored every other day (**c**), and tumor size (**d**) and weight (**e**) are shown. Data are presented as mean ± SD (*n =* 5). *, *p* < 0.05; **, *p* < 0.01. **f-g.** FOXM1 depletion down-regulates TPX2 expression in vivo. Protein level of TPX2 in tumors were checked by western blot (**f**) and IHC (**g**). **h.** Huh7 cells transfected with shRNA-control or shRNA-FOXM1 were incubated with or without N-Shh conditional medium for 48 h and were harvested for WB analysis with the indicated antibodies. **i.** Huh7 cells stably infected with Lv-control or Lv-GLI2A were infected twice with shRNA-control or shRNA-FOXM1 and were harvested for WB with the indicated antibodies. **j.** Hep3B cells transfected with Lv-control or Lv-FOXM1 were treated with DMSO or 20 μM GANT61 for 48 h and were harvested for WB analysis with the indicated antibodies. Data was shown as mean ± SD (*n =* 3). *, *p* < 0.05; **, *p* < 0.01

Next, we investigated the regulation of TPX2 expression by FOXM1. In Huh7 and Hep3B cells, FOXM1 knockdown significantly reduced in both TPX2 protein and mRNA levels (Fig. 5a and S6a), which was in clear contrast to the up-regulated protein and mRNA levels of TPX2 in Huh7 and HepG2 cells over-expressing FOXM1 (Fig. 5b and S6b). Furthermore, we established xenograft HCC tumor models by employing stable doxycycline-inducible Huh7 cell lines that express Lentivirus-shFOXM1. And we found that while knocking down FOXM1, the tumor growth was suppressed (Fig. 5c-e), and at the same time, the expression of TPX2 was also decreased in vivo (Fig. 5f-g). Based on these results, we concluded that FOXM1 is a direct transcriptional activator of TPX2. Furthermore, the Hh-FOXM1-TPX2 signaling was analyzed. In response to treatment with N-Shh condition medium and GLI2A over-expression, the protein levels of both FOXM1 and TPX2 were increased, although the increase in TPX2 levels was significantly reversed by FOXM1 knockdown (Fig. 5h-i), whereas FOXM1 over-expression rescued TPX2 down-regulation after GANT61-mediated inhibition of GLI2 (Fig. 5j). These results revealed the novel Hh-FOXM1-TPX2 signaling pathway in HCC cells.

### TPX2 was a key effector of the pro-proliferative function of FOXM1

**Fig. 6:**
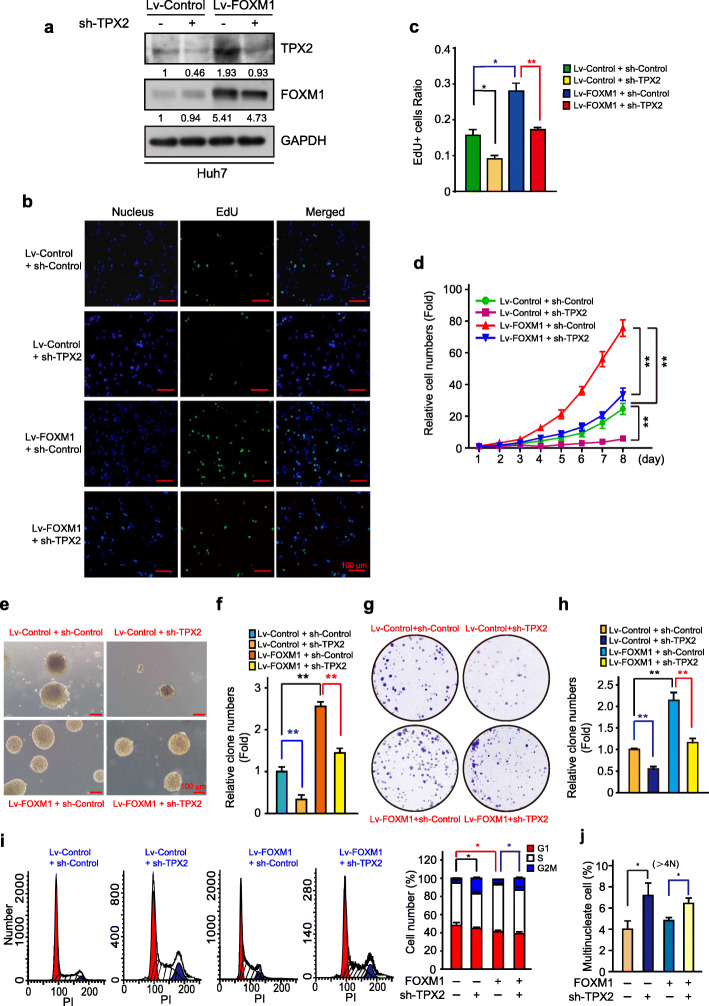
FOXM1 promotes HCC cell proliferation through TPX2. **a.** WB analysis of Huh7 cells infected with FOXM1 overexpression and TPX2 knockdown lentiviral vectors. **b.** Comparison of the proliferative ability of Lv-control + sh-Con, Lv-control + sh-TPX2, Lv-FOXM1 + sh-Con, and Lv-FOXM1 + sh-TPX2 in Huh7 cells using the EdU assay. Scale bar, 100 μm. **c.** The ratio of EdU-positive cells was quantified using the ImageJ software (*n =* 3). **d.** Growth curves of Lv-control + sh-Con, Lv-control + sh-TPX2, Lv-FOXM1 + sh-Con, and Lv-FOXM1 + sh-TPX2 of Huh7 cells. **e-h.** Comparison of the proliferative ability (**f**) and plate colony formation assay (**h**) of Lv-control + sh-Con, Lv-control + sh-TPX2, Lv-FOXM1 + sh-Con, and Lv-FOXM1 + sh-TPX2 in Huh7 cells. Scale bar, 100 μm. Soft agar formation (**g**) and plate colony formation (**I**) were quantified using the ImageJ software. **i.** Huh7 cells stably expressing FOXM1 + shTPX2 were subjected to cell cycle analysis (left panel) and fraction of cells in each phase quantified (right panel). **j.** Quantification (%) of multinucleated Huh7 cells stably expressing FOXM1 + shTPX2. Data was represented as mean ± SD of three independent experiments. *, *p* < 0.05; **, *p* < 0.01

To directly elucidate the epistatic relationship between FOXM1 and TPX2 in HCC proliferation, we weakened the expression of TPX2 in Huh7 cells over-expressing FOXM1 (Fig. 6a) and conducted a series of EdU assays, colony formation assay, and anchorage-independent colony formation and cell survival assays. In agreement with the results of previous reports, FOXM1 expression led to high proportion of EdU-positive cells (Fig. 6b-c), rapid cell growth (Fig. 6d) and enhanced colony formation (Fig. 6e-h). Nevertheless, TPX2 deletion dramatically abrogated HCC proliferation promoted by FOXM1 over-expression (Fig. 6b-h). To further investigate the effects of TPX2 knockdown on cell cycle progression and determine whether TPX2 is a downstream effector in FOXM1-promoted cell cycle progression, we utilized an image-flow cytometry assay to analyze cell cycle distribution and DNA content in Huh7 cells. Results showed higher proportion of polyploid cells (> 4 N) and a cell cycle arrest event in the G2/M phase (Fig. 6i-j). Collectively, these data are consistent with a model in which FOXM1 drives HCC proliferative by regulating TPX2 expression, which is an essential component of the proliferative state.

### Elevated FOXM1 and TPX2 levels are indicators of poor survival for patients with HCC

**Fig. 7:**
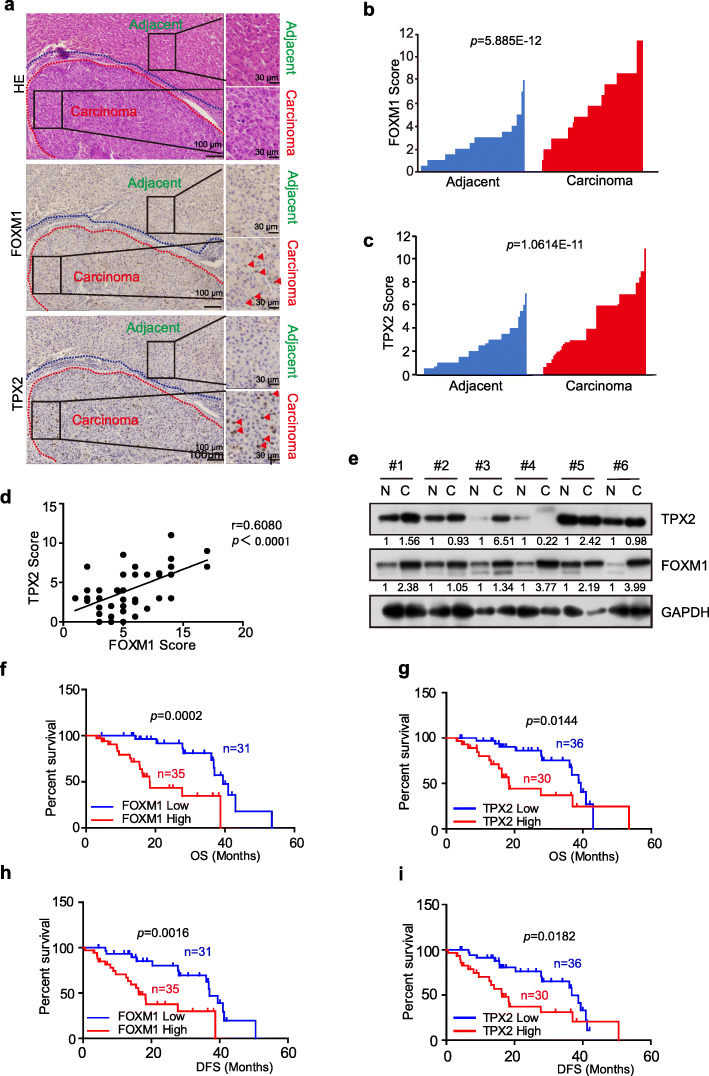
High TPX2 levels correlates with high FOXM1 levels in HCC tumors and with poor survival in patients with HCC. **a**. Representative photomicrographs of H&E-stained liver tissues and IHC staining of FOXM1 and TPX2 in a human HCC sample and the matched adjacent normal tissue sample in the same section. The areas of carcinoma and adjacent normal tissues were sketched. The subcellular locations of FOXM1 and TPX2 are indicated by red arrows. **b**-**c**. FOXM1(**b**) and TPX2(**c**) expression levels were graded using the immunohistochemical scores. Data were analyzed using the Wilcoxon matched-pairs signed-rank test. **d**. Correlation between FOXM1 and TPX2 expression in 66 HCC immunohistochemical samples was plotted as a scatter diagram. Data were analyzed using the Spearman correlation test. **e**. Protein level of TPX2 and FOXM1 in 6 randomly selected HCC samples were checked via immunoblotting. **f-g**. Kaplan-Meier overall survival rate analysis of 66 HCC patients grouped by IHC-assessed FOXM1 (**e**) and TPX2 (**f**) levels. **h**-**i**. Kaplan-Meier disease-free survival rate analysis of 66 HCC patients grouped by IHC-assessed FOXM1 (**g**) and TPX2 (**h**) levels

As TPX2 is a target of FOXM1, we attempted to further clarify the roles of FOXM1 and TPX2 in human HCC samples. We determined the protein levels of FOXM1 and TPX2 in 66 pairs of primary HCC and matched adjacent non-malignant liver tissues using IHC. The IHC analysis of 66 patient samples also confirmed the overexpression of TPX2 and FOXM1 in tumor tissues (Fig. 7a-c). This observation were further confirmed by immunoblotting in in 6 pairs of randomly selected HCC samples, and in most cases, TPX2 and FOXM1 showed higher expression in tumor tissues (Fig. 7e). Owing to the small scale of our clinical cohort study, bioinformatics tools were assisted to determine the significance of our analyzed data. We searched in the public UALCAN website [35] to acquire clinical data from The Cancer Genome Atlas (TCGA) dataset (http://cancergenome.nih.gov/). Results verified that primary HCC tissue showed higher expression of FOXM1 and TPX2 than normal tissue (Fig. S7a-b) in TCGA dataset. All these results validated that FOXM1 and TPX2 are aberrantly highly-expressed in primary HCC samples.

Next, we determined the correlation between FOXM1 and TPX2 expression levels and clinicopathological features of HCC patients. As shown in Table 1, higher levels of both FOXM1 and TPX2 correlated positively with larger tumors, poorer histological grade and later tumor-node-metastasis (TNM) stage. This was in agreement with the results in the public website UALCAN (Fig. S7b). Furthermore, the Spearman correlation coefficient showed that FOXM1 expression correlated positively with that of TPX2 (Fig. 7d), which was in agreement with the results of data analysis obtained using TCGA (Fig. S7c). In addition, cell lines that expressed high levels of FOXM1 also showed higher TPX2 expression (Fig. S7d-e).

To determine whether FOXM1 and TPX2 were associated with the clinical outcomes of patients with HCC, we performed a Kaplan-Meier survival analysis of HCC patients grouped by immunochemistry-assessed FOXM1 and TPX2 protein levels. This analysis revealed that higher FOXM1 and TPX2 protein levels were both significantly associated with poorer overall survival and disease-free survival in HCC (Fig. 7f-i). Our analysis in UALCAN also strongly supported this observation (Fig. S7f-g). Furthermore, the publicly available database Kaplan Meier-plotter validated that high FOXM1 and TPX2 mRNA levels were indicative of poor progression-free survival in two different cohorts of patients with HCC (Fig. S7h-i). These results suggested that FOXM1 and TPX2 are novel negative prognostic markers of HCC.

## Discussion

Although aberrantly activated Hh signaling in HCC had been observed for decades [36], the exact mechanisms how Hh signaling regulates tumor growth remain further study. Vismodegib, a SMO antagonist, had been investigated in a Phase II clinical trial for solid tumors therapy, including HCC (ClinicalTrials.gov Identifier: NCT02465060). As reported in basal cell carcinoma (BCC), tumor cells would frequently acquire resistance to Vismodegib, with multiple SMO mutations [37, 38]. Therefore, it is urgent to understand the regulatory mechanism of Hh signaling pathway activation that occurs downstream of SMO, and to develop multiple target therapies for HCC. Herein, we screened a series of candidate target genes regulated by Hh signaling (Fig. 1), thus to broaden our insights into Hh signaling related HCC.

TPX2 is a well-established oncogene with high expression level in varieties of tumors, including HCC [21], pancreatic cancer [39], and esophageal squamous carcinoma [40], the regulatory mechanism of its expression, however, is not clear yet. We identified TPX2 as a novel downstream target of Hh pathway. The regulation of TPX2 by Hh signaling was observed in multiple HCC cell lines. Suppression of Hh signaling by either small molecule inhibitors or GLI2 knockdown led to the decrease of TPX2 expression, and to the contrary, Hh signaling activation by N-Shh conditional medium or GLI2A over-expression stimulated TPX2 expression (Fig. 1). Inhibition of Hh signaling led to the growth attenuation of HCC cells, however, cells with TPX2 over-expression were resistant to Hh inhibition (Fig. 2). Consistently, cells with TPX2 knockdown had no response to N-Shh stimulation (Fig. 3), indicating that Hh signaling facilitates HCC cell growth via regulating TPX2.

Targeting of the mitotic spindle checkpoint, which induces massive aneuploidy and severe chromosome segregation errors, is being considered for developing new therapeutic strategies for selectively eliminating highly proliferative cancer cells [41–44]. Since TPX2 plays an essential role in mitotic spindle apparatus and subsequently cell division and tumor growth via binding to Aurora-A, many researchers focus on identifying antagonists to blockade this interaction [45]. Our findings of the regulation of TPX2 expression by Hh signaling provide a novel approach to suppress TPX2 via decreasing its expression level rather than its activity. Moreover, combination of suppressing the TPX2 activity and interrupting its expression would further arrest the proliferation of cancer cells, which offers new therapy strategies.

Interestingly, TPX2 was an indirect target gene of GLI, and further study showed that FOXM1, a transcriptional factor activated by Hh/GLI signaling [7], was illustrated to participate in the regulation (Fig. 5). More importantly, TPX2 knockdown attenuated the cell proliferation-promoting ability of FOXM1, which indicated that FOXM1 drives HCC proliferation by regulating TPX2 expression (Fig. 6). These results concluded that TPX2 is regulated by Hh-GLI-FOXM1 signal axis in HCC cells; and it is responsible for cell proliferation and tumor growth induced by this signal axis. Additionally, our clinical cohort study of 66 HCC patients further proved that TPX2, together with FOXM1, are highly expressed in HCC tissues, and are significantly correlated. Higher expression of either TPX2 or FOXM1 is related to poorer prognosis of HCC patients, which had been illuminated in both our own clinical cohort and TCGA cohort (Fig. 7). These results further supported our findings on Hh-FOXM1-TPX2 signaling axis which regulates cell proliferation and tumor growth in HCC.

Nevertheless, some result showed that depletion of TPX2 did not rescue the function of Hh signaling (Fig. S3) or FOXM1 completely (Fig. 6), though their effect were dramatically reduced. This might was led by two possible reasons: firstly, knockdown TPX2 might not fully depletion the function of TPX2, and the remnant TPX2 might mediate the function of FOXM1; secondly, FOXM1, as well as Hh signaling, has varieties of downstream target genes, some of which might mediates its regulation in cell proliferation.

Inhibition of FOXM1 activity is attractive for cancer therapy and several small molecule inhibitors were identified to suppress FOXM1 through multiple mechanisms [46]. The majority of FOXM1 antagonists, such as thiostrepton [47], honokiol [48], FDI-6 [49] and FOXM1 Apt [50], abrogate to the binding of FOXM1 to target sites, thus to restrict the expression of its downstream target genes. We believed that united medication with Hh inhibition and FOXM1 attenuation would improve the efficiency and delay drug resistance, but more evidence should be carefully collected for this novel strategy. Additionally, specific diarylheptanoids were reported to suppress proliferation of pancreatic cancer cells through modulating Hh-GLI-FOXM1 pathway [51], indicating a promising approach to blockade Hh-GLI-FOXM1 signal axis specifically. Even though, many potential questions still exist. For instance, in terms of mechanism, whether the Hh-FOXM1-TPX2 axis also participates in other biological processes of HCC? In practical applications, does combination therapy of these inhibitors bring about certain unexpected side effects? Answering these questions will contribute to acquire a better understanding of HCC biology, which can subsequently assist in the development of new modalities for cancer treatment.

## Conclusions

In conclusion, we reported a novel signal axis through which Hh signaling regulates tumor growth via FOXM1 and TPX2. Hh signaling activates the transcription factor GLI2, which directly transactivates FOXM1. FOXM1 then induces transcriptional upregulation of TPX2 to promote G2/M phase progression and HCC cell proliferation. Inhibition of the Hh-FOXM1-TPX2 axis can be utilized to suppress HCC cell proliferation, and each network node in this axis could be a promising target for HCC therapy.

## Supplementary information

**Additional file 1: Supplementary Figures 1-6.SupplementaryFig. 1.** a. Function clustering of DEGs. b-c. Huh7 (b) and HepG2 (c) cells were treated with GANT61 (20 μM) for 48 h and harvested for real-time PCR analysis with the indicated primers. d-e. Previous Hh target screening via microarray. f-g. LM-3 cells were cells were treated with GANT61 (f) and Cyclopamine (g) for 48 h and harvested for real-time PCR analysis with the indicated primers. Tomatidine was used as control for Cyclopamine. h-i. Huh7 (h) and HepG2 (i) cells were treated with Cyclopamine (20 μM) for 48 h and harvested for real-time PCR analysis with the indicated primers. Tomatidine was used as control. Data was shown as mean ± SD (*n* = 3). *, *p* < 0.05; **, *p* < 0.01. **Supplementary Fig. 2.** a. Validation of SK-Hep-1 cells stably over-expressing TPX2 using WB analysis with the indicated antibodies. b. Comparison of the proliferative ability of Lv-control + DMSO, Lv-control + GANT61 (2 μM), Lv-TPX2 + DMSO, and Lv-TPX2 + GANT61 (2 μM) in SK-Hep-1 cells treated with EdU. Scale bar, 100 μm. c. The ratio of EdU-positive cells was quantified using the ImageJ software (*n =* 3). d. Cell growth curves of Lv-control + DMSO, Lv-control + GANT61 (2 μM), Lv-TPX2 + DMSO, and Lv-TPX2 + GANT61 (2 μM) in SK-Hep-1 cells. e-f. Comparison of the proliferative ability of Lv-control + DMSO, Lv-control + GANT61 (2 μM), Lv-TPX2 + DMSO, and Lv-TPX2 + GANT61 (2 μM) in HepG2 cells using colony formation assay. Colonies were counted using the ImageJ software. Data was shown as mean ± SD (*n =* 3). *, *p* < 0.05; **, *p* < 0.01, N.S. denotes not significant. **Supplementary Fig. 3.** a. Validation of Hep3B sh-TPX2 stable cell lines using WB analysis. b. TPX2 abrogation inhibited Hh signaling-induced HCC cell proliferation as determined using EdU staining. Scale bar, 100 μm. c. The ratio of EdU-positive cells was quantified using the ImageJ software (*n =* 3). d. Cell growth curves of sh-Control / Shh (−), sh-Control / Shh (+), sh-TPX2 / Shh (−), and sh-TPX2 / Shh (+) in Hep3B cells. e-f. Comparison of the proliferative ability of sh-Control / Shh(−), sh-Control / Shh (+), sh-TPX2 / Shh(−), and sh-TPX2 / Shh (+) in Hep3B cells using colony formation assay. Colonies were counted using the ImageJ software. g. Marker of cell proliferation, PCNA, were determined via WB analysis. Data was shown as mean ± SD (*n =* 3). *, *p* < 0.05; **, *p* < 0.01. **Supplementary Fig. 4.** a. Validation of Huh7 sh-TPX2 stable cell lines using quantitative real-time PCR. b. TPX2 abrogation inhibited Hh signaling-induced HCC cell proliferation as determined using EdU staining. Scale bar, 100 μm. c. The ratio of EdU-positive cells was quantified using the ImageJ software (*n =* 3). **Supplementary Fig. 5.** a-d. The expression of FOXM1 in Fig. 4d (a), 4e (b), 4f (c) and 4 g (d) were testified via immunoblotting. **Supplementary Fig. 6.** a. Real-time PCR to determine the mRNA levels of TPX2 and FOXM1 in Huh7 and Hep3B cells with stable knockdown of FOXM1. b. Real-time PCR analysis to determine the mRNA levels of TPX2 and FOXM1 in Huh7 and HepG2 cells stably over-expressing FOXM1. **Supplementary Fig. 7.** a. Expression of FOXM1 and TPX2 in HCC tissue and normal tissue based on TCGA. b. Expression of FOXM1 and TPX2 in HCC based on tumor grade in UALCAN database. **, *p*<0.01. c. Correlation analysis of TPX2 and FOXM1 mRNA levels in TCGA database (*n =* 381). d-e. Five human HCC cell lines showed varying protein (d) and mRNA (e) expression of FOXM1 and TPX2. f-g. Kaplan-Meier overall survival rate analysis of a set of HCC cancer patients grouped by FOXM1 (f) and TPX2 (g) levels in TCGA dataset. h-i. Kaplan-Meier progression-free survival rate analysis of a set of HCC cancer patients grouped by FOXM1 (h) and TPX2 (i) levels in the Kaplan-Meier Plotter website.

**Additional file 2: Table S1.** Target sequences of gene-silencing constructs. **Table S2.** Primers for Real-time PCR amplification. **Table S3.** Primer sequences for TPX2-Luciferase reporter constructs. **Table S4.** Primer for ChIP.

**Additional file 3: Table S5.** Function clustering of DEGs.

## Data Availability

All data generated or analyzed during this study are included in this published article and its supplementary information files. Raw data of RNA-Seq is available at sequence read archive (SRA) with accession no. PRJNA592618 and raw data of microarray is available at GEO with accession no. GSE73481.

## Abbreviations

ANOVA: analysis of variance
AJCC: American Joint Committee on Cancer
BS: Binding site
ChIP: Chromatin immunoprecipitation
DMEM: Dulbecco’s Modified Eagle’s Medium
EdU: 5-ethynyl-2′-deoxyuridine
EMT: Epithelial-mesenchymal transition
FOX: Forkhead Box
FOXM1: Forkhead box M1
GAPDH: Glyceraldehyde 3-phosphate dehydrogenase
GEO: Gene Expression Omnibus
HBV: Hepatitis B virus
HCC: Hepatocellular carcinoma
H&E: Hematoxylin and eosin
Hh: Hedgehog
IHC: Immunohistochemistry
IARC: International Agency for Research on Cancer
MEM: Minimum Essential Medium
PBS: Phosphate-buffered saline
PI: propidium iodide
PTCH1: Patched-1
shRNA: short hairpin RNA
SHH: Sonic Hh
SMO: Smoothened
TCGA: The Cancer Genome Atlas
TNM: Tumor-node-metastasis
TPX2: Targeting protein for Xenopus kinesin-like protein 2
