# Supplementary material for: The critical role of dysregulated Hh-FOXM1-TPX2 signaling in human hepatocellular carcinoma cell proliferation
Source: Cell Commun Signal. 2020 Jul 28;18:116. doi: 10.1186/s12964-020-00628-4 (PMC7388463; doi:10.1186/s12964-020-00628-4)

Supplementary Figure 1

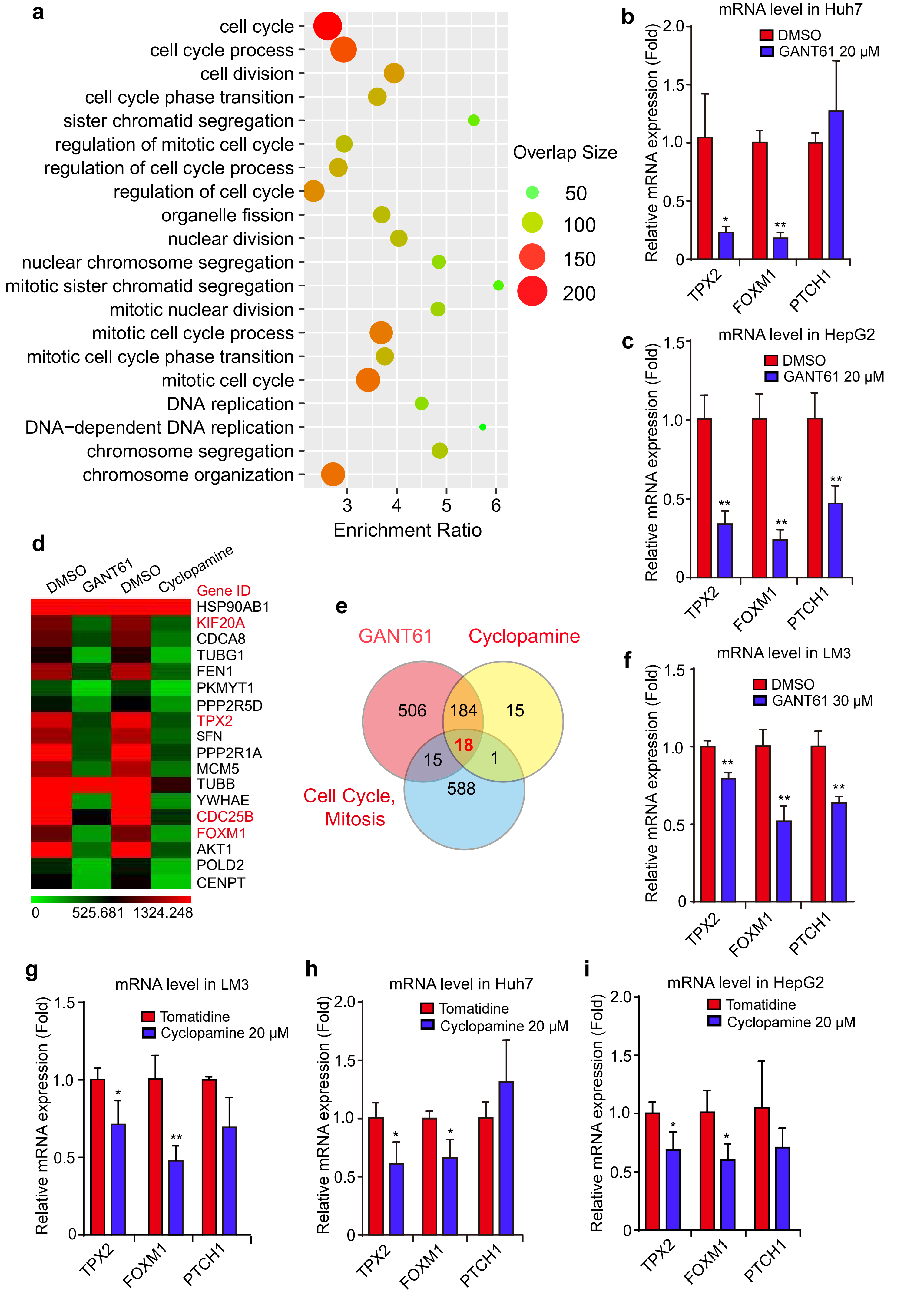

Supplementary Figure 2

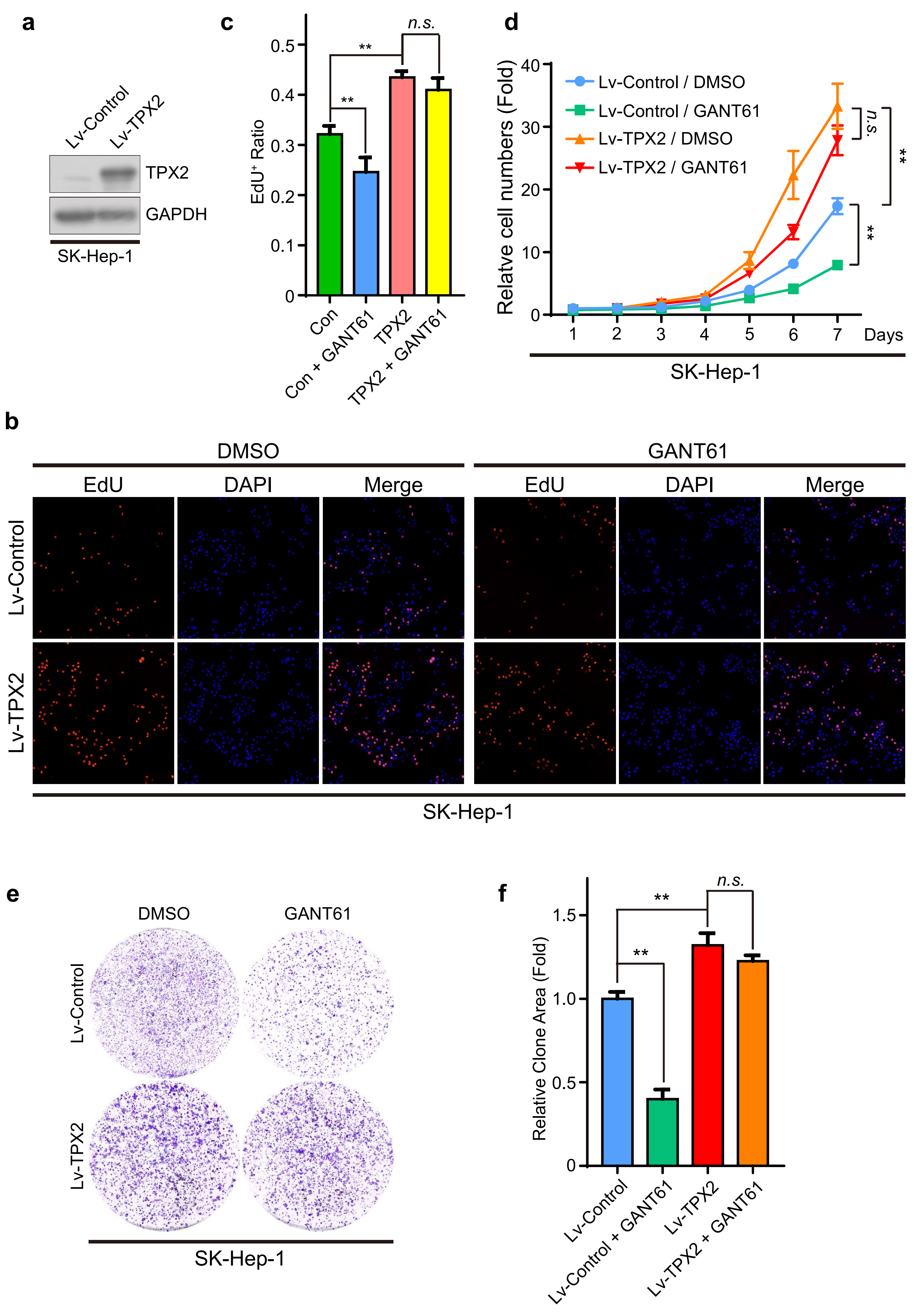

Supplementary Figure 3

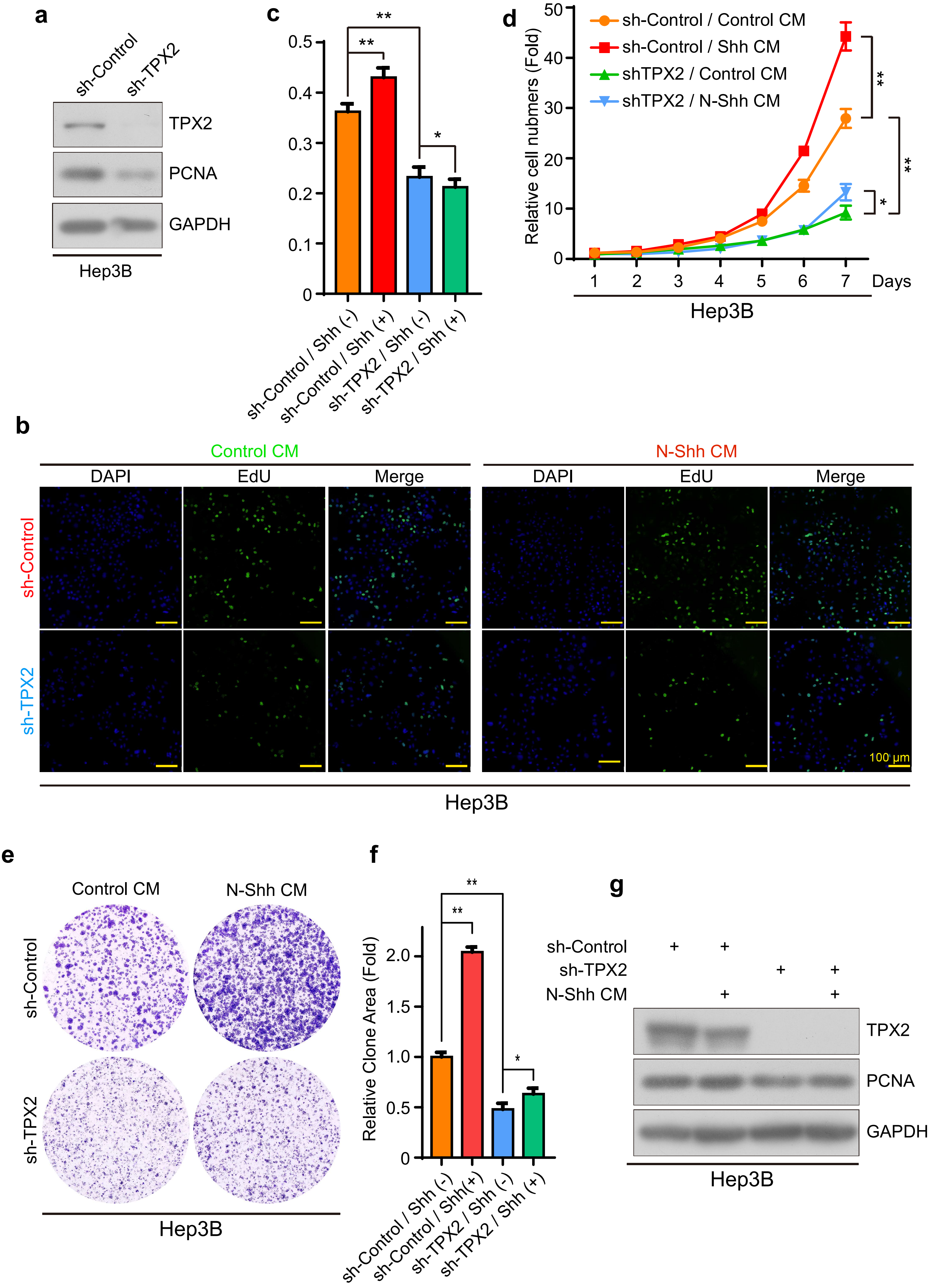

Supplementary Figure 4

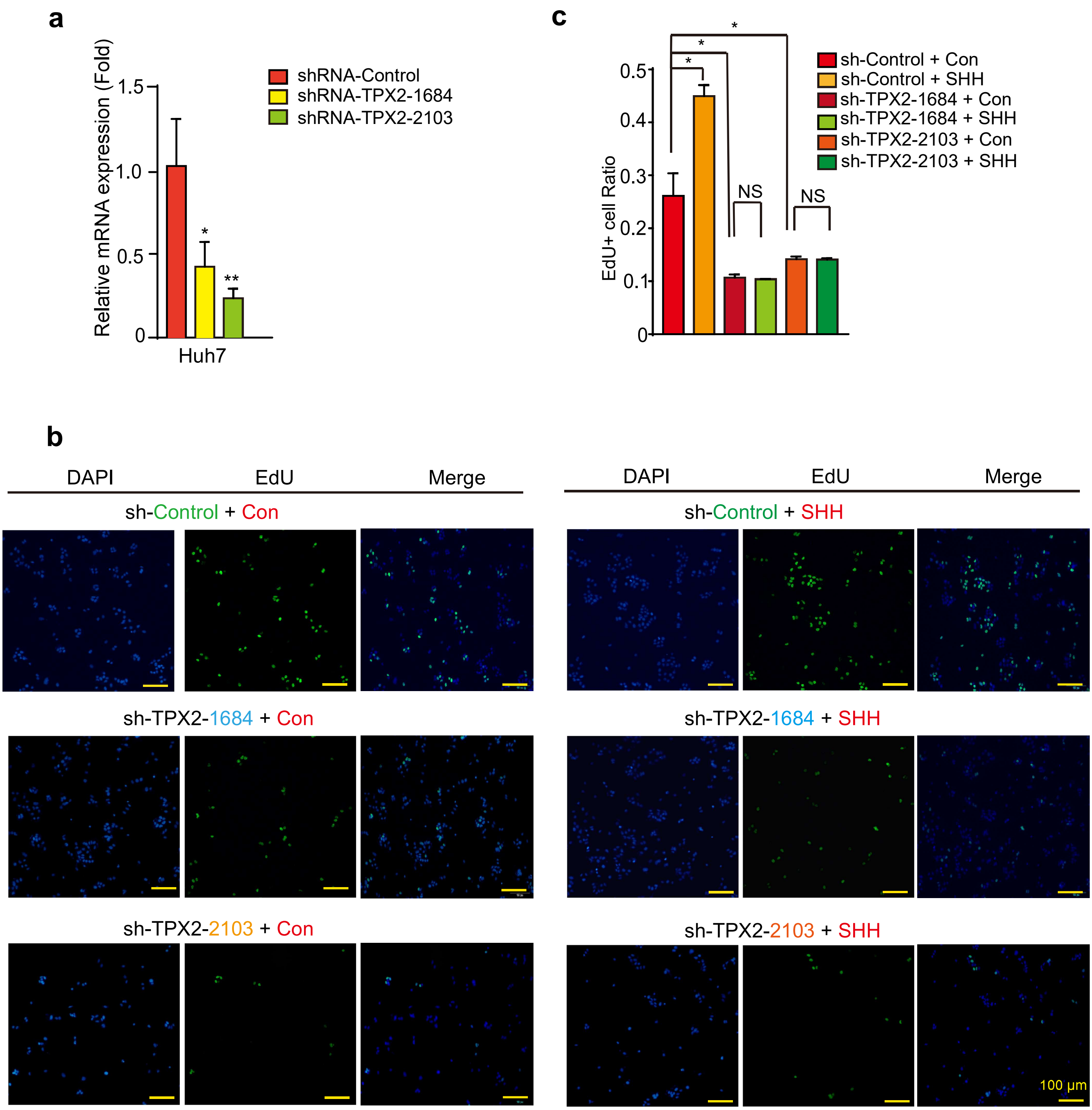

Supplementary Figure 5

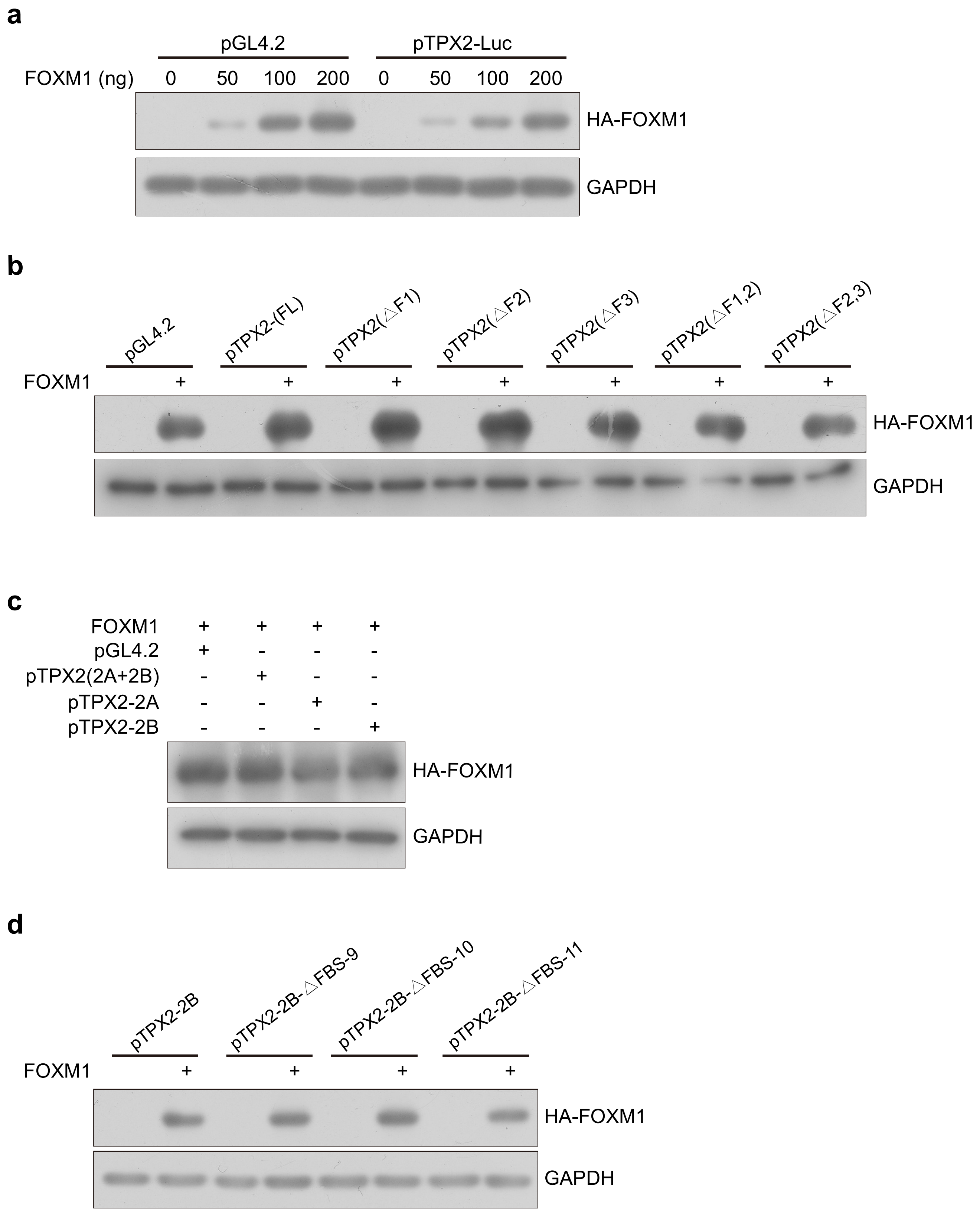

Supplementary Figure 6

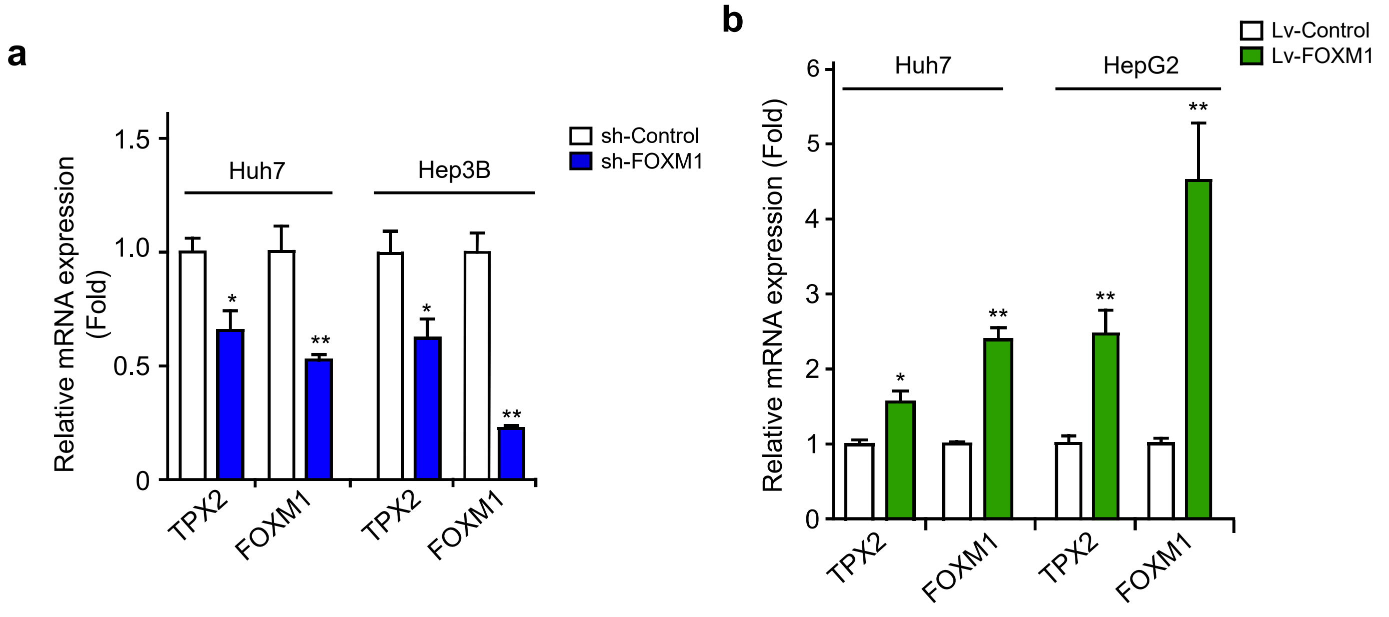

Supplementary Figure 7

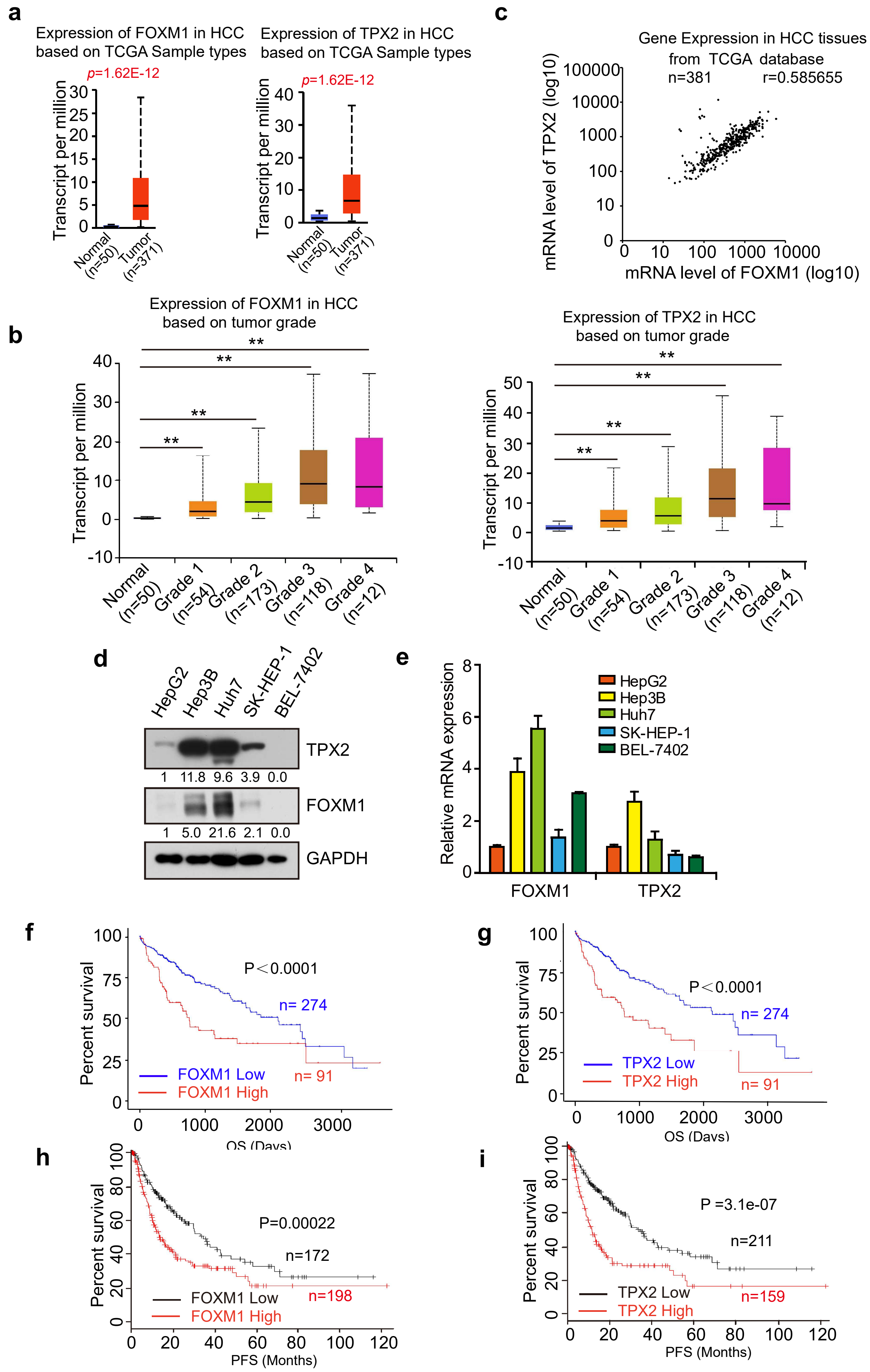

Supplement: Supplementary file 1 — Additional file 1: Supplementary Figures 1-6.SupplementaryFig. 1. a. Function clustering of DEGs. b-c. Huh7 (b) and HepG2 (c) cells were treated with GANT61 (20 μM) for 48 h and harvested for real-time PCR analysis with the indicated primers. d-e. Previous Hh target screening via microarray. f-g. LM-3 cells were cells were treated with GANT61 (f) and Cyclopamine (g) for 48 h and harvested for real-time PCR analysis with the indicated primers. Tomatidine was used as control for Cyclopamine. h-i. Huh7 (h) and HepG2 (i) cells were treated with Cyclopamine (20 μM) for 48 h and harvested for real-time PCR analysis with the indicated primers. Tomatidine was used as control. Data was shown as mean ± SD (n = 3). *, p < 0.05; **, p < 0.01. Supplementary Fig. 2. a. Validation of SK-Hep-1 cells stably over-expressing TPX2 using WB analysis with the indicated antibodies. b. Comparison of the proliferative ability of Lv-control + DMSO, Lv-control + GANT61 (2 μM), Lv-TPX2 + DMSO, and Lv-TPX2 + GANT61 (2 μM) in SK-Hep-1 cells treated with EdU. Scale bar, 100 μm. c. The ratio of EdU-positive cells was quantified using the ImageJ software (n = 3). d. Cell growth curves of Lv-control + DMSO, Lv-control + GANT61 (2 μM), Lv-TPX2 + DMSO, and Lv-TPX2 + GANT61 (2 μM) in SK-Hep-1 cells. e-f. Comparison of the proliferative ability of Lv-control + DMSO, Lv-control + GANT61 (2 μM), Lv-TPX2 + DMSO, and Lv-TPX2 + GANT61 (2 μM) in HepG2 cells using colony formation assay. Colonies were counted using the ImageJ software. Data was shown as mean ± SD (n = 3). *, p < 0.05; **, p < 0.01, N.S. denotes not significant. Supplementary Fig. 3. a. Validation of Hep3B sh-TPX2 stable cell lines using WB analysis. b. TPX2 abrogation inhibited Hh signaling-induced HCC cell proliferation as determined using EdU staining. Scale bar, 100 μm. c. The ratio of EdU-positive cells was quantified using the ImageJ software (n = 3). d. Cell growth curves of sh-Control / Shh (−), sh-Control / Shh (+), sh-TPX2 / Shh (−), [file 12964_2020_628_MOESM1_ESM.pdf]
