# Supplementary material for: The critical role of dysregulated Hh-FOXM1-TPX2 signaling in human hepatocellular carcinoma cell proliferation
Source: Cell Commun Signal. 2020 Jul 28;18:116. doi: 10.1186/s12964-020-00628-4 (PMC7388463; doi:10.1186/s12964-020-00628-4)
Supplement: Supplementary file 2 — Additional file 2: Table S1. Target sequences of gene-silencing constructs. Table S2. Primers for Real-time PCR amplification. Table S3. Primer sequences for TPX2-Luciferase reporter constructs. Table S4. Primer for ChIP. [file 12964_2020_628_MOESM2_ESM.docx]

**Supplementary Table**

**Table S1. Target sequences of gene-silencing constructs**

| Name | Target sequences (5' to 3') |
| --- | --- |
| shRNA-FOXM1-109 | 5'-GCCCAACAGGAGTCTAATCAA-3' |
| shRNA-FOXM1-731 | 5'-TGATACAATTCGCCATCAACA-3' |
| shRNA-FOXM1-1692 (*) | 5'-TGTCTCGGAAATGCTTGTGAT-3' |
| shRNA-TPX2-1684 (*) | 5'-AGCAAGTTGAAGACTTCCATA-3' |
| shRNA-TPX2-2103 | 5'-GATGTTGTGGGTGTTCCTGAA-3' |
| shRNA-TPX2-2178 | 5'-AGAATTCGAATGCCCACCAAA-3' |

**Notes:**

*: Sequence For Lentivirus shRNA System.

**Table S2: Primers for Real-time PCR amplification**

| Primer name | Sequence |
| --- | --- |
| GLI2-Forward | 5'-CCACCACCTCACCCAGTCCA-3' |
| GLI2-Reverse | 5'-CAAAGCCTGCTGTAGCCACCC-3' |
| PTCH1-Forward | 5'-ACTCCCAAGCAAATGTACGAG-3' |
| PTCH1-Reverse | 5'-TTGAGTGGAGTTCTGTGCG-3' |
| FOXM1-Forward | 5'-ACCGCTACTTGACATTGGAC-3' |
| FOXM1-Reverse | 5'-GGGAGTTCGGTTTTGATGGTC-3' |
| TPX2-Forward | 5'-GAAGAGAATGGCTGAGGTAGAAG-3' |
| TPX2-Reverse | 5'-CTGGTACTTGCGTATTGGATTTG-3' |
| GAPDH-Forward | 5'-AATCCCATCACCATCTTCCAG-3' |
| GAPDH-Reverse | 5'-AAATGAGCCCCAGCCTTC-3' |

**Table S3. Primer sequences for TPX2-Luciferase reporter constructs**

| Primer name | | | Sequence (5' to 3') |
| --- | --- | --- | --- |
| FL-Forward | | 5'-CAACTACCACTCCCCCATCC-3' | |
| FL-Reverse | | 5'-ACTCACCCAAACCAACCAGG-3' | |
| Δ1-Forward | | 5'-TACATGCGTGAGCTACCGCACCCAG-3' | |
| Δ1-Reverse | | 5'-GGAGTGGTAGTTGTTGTGGTGGTTG-3' | |
| Δ2-Forward | | 5'-TAGGCCTGGCTTCTGAGGCGGTTG-3' | |
| Δ2-Reverse | | 5'-ATCCCAGCACTTTGGGAGGCCGAG-3' | |
| Δ3-Forward | | 5'-CCCTGGTTGGTTTGGGTGAGTCAG-3' | |
| Δ3-Reverse | | 5'-CTCGCCTCAGATCTGACAACGAAG-3' | |
| Fragment 2A-Forward | 5'-CCGCTCGAGGGACATGTTTGTTCAACTTCTCCTAG-3' | | |
| Fragment 2A-Reverse | 5'-CCCAAGCTTGCATCCTGGACCTCTCCAAGTT-3' | | |
| Fragment 2B-Forward | 5'-CCGCTCGAGAACTTGGAGAGGTCCAGGATGC-3' | | |
| Fragment 2B-Reverse  BS9 Mut-Forward  BS9 Mut-Reverse  BS10 Mut-Forward  BS10 Mut-Reverse  BS11 Mut-Forward  BS11 Mut-Reverse | 5'-CCCAAGCTTTCAGATCTGACAACGAAGCGCGA-3'  5'-TGCCCTCCACCTCTTCAGTTGGTAC-3'  5'-ACCGGAGATCACAGAATGGCGCG-3'  5'-ATTCGGAGCAGAGCACTCCGGTC-3'  5'-TGTAGGGGAAAGGTGGTACCAACTG-3'  5'-AGTCCCCGGCGCTCTGATTGGTG-3'  5'-CCAGGCCAGGCTCCCGATTGGCC-3' | | |

**Notes:**

BS: Binding Site.

**Table S4. Primer for ChIP**

| Primer name | Sequence (5' to 3') |  |
| --- | --- | --- |
| BS6-Forward  BS6-Reverse | 5'-GGGATGCAGTTGGGAATA-3'  5'-ACATAGACTCGCCCGTAG-3' | |
| BS7-Forward  BS7-Reverse | 5'-TATTGGGAAGGACGCTAC-3'  5'-CTTGAAGGTTCAGGGAGT-3' | |
| BS8-Forward  BS8-Reverse | 5'-CAAGGGATAATGCACAAC-3'  5'-CCTAGATCACAGAATGGC-3' | |
| BS9-Forward  BS9-Reverse | 5'-TTTCTTCCCTGAAAACTTGGA-3'  5'-GGAGTGCTCTGCTCCAAACT-3' | |
| BS10-Forward  BS10-Reverse | 5'-TGGTACCACCTTTCCCAAAT-3'  5'-GCCTGCGTGAGTCCACTG-3' | |
| BS11-Forward  BS11-Reverse | 5'-GTATTGCTGCGGTCTGTAG-3'  5'-GGGACTCATTCAGTATCGT-3' | |

**Notes:**

BS: Binding Site.
